# The hidden financial catastrophe of chronic kidney disease under universal coverage and Thai “Peritoneal Dialysis First Policy”

**DOI:** 10.3389/fpubh.2022.965808

**Published:** 2022-10-13

**Authors:** Pornpen Sangthawan, Pinkaew Klyprayong, Sarayut L. Geater, Pimwara Tanvejsilp, Sirirat Anutrakulchai, Sarinya Boongird, Pongsathorn Gojaseni, Charan Kuhiran, Pichet Lorvinitnun, Kajohnsak Noppakun, Watanyu Parapiboon, Supinda Sirilak, Pluemjit Tankee, Puntapong Taruangsri, Pasuree Sangsupawanich, Piyamitr Sritara, Nathorn Chaiyakunapruk, Chagriya Kitiyakara

**Affiliations:** ^1^Department of Medicine, Faculty of Medicine, Prince of Songkla University, Songkhla, Thailand; ^2^Department of Medicine, Faculty of Medicine Ramathibodi Hospital, Mahidol University, Bangkok, Thailand; ^3^Department of Pharmacy Administration, Faculty of Pharmaceutical Sciences, Prince of Songkla University, Songkhla, Thailand; ^4^Department of Medicine, Faculty of Medicine, Khon Kaen University, Khon Kaen, Thailand; ^5^Department of Medicine, Bhumibol Adulyadej Hospital, Directorate of Medical Services, Royal Thai Air Force, Bangkok, Thailand; ^6^Department of Medicine, Somdej Pranangchao Sirikit Hospital, Chonburi, Thailand; ^7^Department of Medicine, Sunpasitthiprasong Hospital, Ubon Ratchathani, Thailand; ^8^Department of Internal Medicine, Faculty of Medicine, Chiang Mai University, Chiang Mai, Thailand; ^9^Department of Medicine, Maharat Nakhonratchasima Hospital, Nakhon Ratchasima, Thailand; ^10^Department of Internal Medicine, Naresuan University Hospital, Naresuan University, Phitsanulok, Thailand; ^11^Department of Medicine, Vachiraphuket Hospital, Phuket, Thailand; ^12^Department of Internal Medicine, Nakornping Hospital, Chiang Mai, Thailand; ^13^Department of Pediatrics, Faculty of Medicine, Prince of Songkla University, Songkhla, Thailand; ^14^Department of Pharmacotherapy, College of Pharmacy, University of Utah, Salt Lake City, UT, United States; ^15^IDEAS Center, Veterans Affairs Salt Lake City Healthcare System, Salt Lake City, UT, United States

**Keywords:** Asia, catastrophic health expenditure, economic, kidney failure, impoverishment, universal health insurance

## Abstract

**Objective:**

Universal health coverage can decrease the magnitude of the individual patient's financial burden of chronic kidney disease (CKD), but the residual financial hardship from the patients' perspective has not been well-studied in low and middle-income countries (LMICs). This study aimed to evaluate the residual financial burden in patients with CKD stage 3 to dialysis in the “PD First Policy” under Universal Coverage Scheme (UCS) in Thailand.

**Methods:**

This multicenter nationwide cross-sectional study in Thailand enrolled 1,224 patients with pre-dialysis CKD, hemodialysis (HD), and peritoneal dialysis (PD) covered by UCS and other health schemes for employees and civil servants. We interviewed patients to estimate the proportion with catastrophic health expenditure (CHE) and medical impoverishment. The risk factors associated with CHE were analyzed by multivariable logistic regression.

**Results:**

Under UCS, the total out-of-pocket expenditure in HD was over two times higher than PD and nearly six times higher than CKD stages 3–4. HD suffered significantly more CHE and medical impoverishment than PD and pre-dialysis CKD [CHE: 8.5, 9.3, 19.5, 50.0% (*p* < 0.001) and medical impoverishment: 8.0, 3.1, 11.5, 31.6% (*p* < 0.001) for CKD Stages 3–4, Stage 5, PD, and HD, respectively]. In the poorest quintile of UCS, medical impoverishment was present in all HD and two-thirds of PD patients. Travel cost was the main driver of CHE in HD. In UCS, the adjusted risk of CHE increased in PD and HD (OR: 3.5 and 16.3, respectively) compared to CKD stage 3.

**Conclusions:**

Despite universal coverage, the residual financial burden remained high in patients with kidney failure. CHE was considerably lower in PD than HD, although the rates remained alarmingly high in the poor. The “PD First' program” could serve as a model for other LMICs. However, strategies to minimize financial distress should be further developed, especially for the poor.

## Introduction

Chronic kidney disease (CKD) is a leading cause of catastrophic health expenditure (CHE) and impoverishment worldwide (1–3). As CKD progresses to kidney failure, kidney replacement therapy is generally provided through public funds in high-income countries. In low-income countries, government funding is not available, and the high out-of-pocket costs make kidney replacement unaffordable for most people. Kidney replacement in middle-income countries may be provided by combined public and private sources (4, 5). Globally, hemodialysis (HD) is the most widely used kidney replacement modality, although it incurs higher costs as it is usually performed in centers in large cities (3, 4). Continuous ambulatory peritoneal dialysis (PD) requires less infrastructure development as patients are treated at home but are used less frequently. By contrast, transplantation is less commonly performed in low and middle-income countries (LMICs).

Universal health coverage can decrease the magnitude of the individual patient's financial burden of CKD (4, 5), but in LMICs, coverage for kidney replacement is often not included because of the high costs. Thailand is an upper-middle-income country with a population of 70 million. The prevalence of CKD stages 1–5 in Thailand was 8.7% (6), and the number of patients on kidney replacement therapy in 2020 included 129,724 HD patients and 34,467 PD patients (7). In 2002, the Thai government initiated the Universal Coverage Scheme (UCS) to cover previously uninsured subjects outside the other two public schemes: the Social Security Scheme (SSS) for company employees and the Civil Servant Medical Benefit Scheme (CSMBS) (8). In 2008, the UCS coverage was extended to dialysis care in a “PD First Policy,” meaning that all new kidney failure patients must use PD as first-line therapy (9–11). Only patients with contraindications to PD were eligible for reimbursement for the cost of HD. By comparison, both HD and PD are reimbursable under SSS or CSMBS. All healthcare schemes provide coverage for essential medications. With UCS accounting for 75% of the population, kidney replacement coverage in Thailand for all healthcare schemes is 98.5% (8, 12). As such, UCS and the “PD First” program in Thailand has often been used as a successful example of kidney care policy in a resource-limited setting (4, 5, 13).

The core principle of universal coverage means that all people have adequate health services without financial hardship (4, 5). Surveys based on expert opinions have provided valuable data on the costs of kidney replacement to governments around the world (14). Still, the residual financial hardship from the patients' perspective despite universal coverage in LMICs including Thailand remains unknown. The out-of-pocket spending for costs not included in the benefits package may be catastrophic for patients and their families. This study aimed to evaluate the out-of-pocket expenditure, CHE, and impoverishment in CKD stage 3 to dialysis under UCS and the “PD First” strategy in a multicenter nationwide study in Thailand by direct patient interviews. For comparisons, we also studied patients under SSS and CSMBS. This information will provide essential data for policy decision-makers in LMICs contemplating universal coverage for kidney replacement.

## Methods

### Study design

This cross-sectional multicenter nationwide study is reported by following the STROBE Statement (15).

### Data source and target population

We conducted this study in 11 tertiary or regional hospitals covering all five regions in Thailand between June 2019 and January 2021 as part of the CORE-CKD study (TCTR20211209001) (www.thaiclinicaltrials.org). Patients (*n* = 100–200) were randomly selected from each hospital. The study population consisted of four groups of CKD patients aged 18 years or older: CKD 15–60 ml/min/1.73 m^2^ (Stages 3–4), CKD<15 ml/min/1.73 m^2^ (stage 5, but not on dialysis), PD and HD covered by health insurance schemes; Universal Coverage Scheme (UCS), Social Security System (SSS), Civil Servant Monetary Benefit Scheme (CSMBS) (Supplementary Table 1) (12). We excluded patients with incomplete expenditure data or those who entirely self-paid.

### Data collection

We collected demographic and clinical data by interviewing patients and caregivers and reviewing medical charts (Supplementary Questionnaire). The estimated GFR (eGFR) was calculated by the CKD-EPI equation (16). *Socioeconomic data* included patient income, food expenditure, and total household consumption spending within 1 month preceding the interview. O*ut-of-pocket expenditures (OOPE)* within 6 months before the interview were collected and categorized into medical or non-medical. Medical OOPE consisted of co-payments, which the health schemes did not cover. Non-medical OOPE consisted of food, transportation, accommodation during clinic visits and hospital admissions, home renovations or expenses for patients' care. Total annual expenditures were calculated in Thai baht, adjusted with the cumulative inflation rate from the data collection to 2021, and then converted to US dollars using the exchange rate in January 2021.

### Outcomes of interest

Financial hardship was measured by the proportion of patients with Catastrophic Health Expenditure (CHE) as the primary outcome and the proportion of medical impoverishment as the secondary outcome. ***CHE40*** was defined as a condition that patient's health care expenditure was at least 40% of the household's capacity to pay as used by WHO (17). Capacity to pay was defined as the effective income (based on total household expenditure) remaining after subtracting basic subsistence costs. We defined pre-out-of-pocket impoverishment based on total household expenditure below the computed subsistence expenditure before deduction of OOPE for health. *Medical impoverishment* was defined as non-poor households that became poor after OOPE for healthcare services (18, 19).

### Statistical analysis

Categorical data were shown as numbers and percentages and compared using the Chi-squared test or Fisher's exact test. Continuous variables were shown as mean with standard deviations (SD) or median with interquartile range (IQR) and compared using one-way analysis of variance, or Kruskal-Wallis test, as appropriate. The proportion (%) of CHE and medical impoverishment were compared among CKD and health schemes. CHE and medical impoverishment were compared across socioeconomic groups, ranked into quintiles based on the equivalized per capita total household expenditure.

We performed multivariable logistic regression analysis to determine factors affecting CHE controlling for the following covariates: age, gender, types of health schemes, groups of CKD, comorbidities (diabetes, hypertension, dyslipidemia, and cardiovascular disease), annual patient income and the number of household members. We also included the interaction terms between groups of CKD and health schemes in the models. The adjusted probability of CHE among different CKD groups, health insurance schemes and geographic regions was calculated. We also performed variance correction for correlation due to the cluster site.

We performed sensitivity analyses by (1) defining *CHE10* as an OOPE for health over 10% threshold level of total household consumption expenditure (18, 19) or (2) defining impoverishment based on Thailand's National poverty line year 2019 (20). All analyses were performed using STATA 16.1, and statistical significance was set at *p* < 0.05.

## Results

### Patient characteristics

Of initial participants (*n* = 1,239), we excluded two patients with incomplete expenditure data and 13 patients who were entirely self-paid (Supplementary Figure 1). A total of 1,224 patients [CKD15-60 (*n* = 435); CKD<15 (*n* = 213); PD (*n* = 257); HD (*n* = 319)] participated in the study (Table 1). There were 44% under UCS, 9% under SSS and 47% under CSMBS health schemes.

**Table 1:** Demographic and clinical characteristics by CKD groups.

**Characteristics**	**Total (%)**	**CKD15-60 (%)**	**CKD<15 (%)**	**PD (%)**	**HD (%)**
Number of patients	1,224	435	213	257	319
**Demographic data**					
Age (years)^a^	63.8 (14.3)	69.0 (12.2)	65.7 (13.2)	58.2 (14.8)	59.8 (14.3)
Female	538 (44)	170 (39.1)	117 (54.9)	115 (44.7)	136 (42.6)
**Health insurance schemes**					
UCS	540 (44.1)	153 (35.2)	108 (50.7)	185 (72.0)	94 (29.5)
SSS	109 (8.9)	24 (5.5)	15 (7.0)	11 (4.3)	59 (18.5)
CSMBS	575 (47.0)	258 (59.3)	90 (42.3)	61 (23.7)	166 (52.0)
**Clinical characteristics**					
eGFR(ml/min/1.73 m^2^)^b^	8 (5–25)	32 (23–42)	9 (7–13)	4 (4–6)	5 (4–6)
Duration of CKD (months)^b^		48 (22–108)	36 (20–68.5)	N/A	N/A
Duration of dialysis (months)^b^		N/A	N/A	35 (20–61.0)	58 (32–100)
Diabetes	552 (45.1)	203 (46.7)	111 (52.1)	109 (42.4)	129 (40.4)
Hypertension	1,121 (91.6)	377 (86.7)	196 (92.0)	242 (94.2)	306 (95.9)
Cardiovascular disease	188 (15.4)	60 (13.8)	28 (13.1)	44 (17.1)	56 (17.6)
Dyslipidemia	871 (71.2)	339 (77.9)	159 (74.6)	162 (63.0)	211 (66.1)
**Sites of study**					
Provincial hospital	514 (42.0)	164 (37.7)	90 (42.3)	116 (45.1)	144 (45.1)
University hospital	710 (58.0)	271 (62.3)	123 (57.7)	141 (54.9)	175 (54.9)

UCS, Universal Coverage Scheme; SSS, Social Security System; CSMBS, Civil Servant Monetary Benefit Scheme.

CKD15-60 chronic kidney disease with eGFR 15-60 ml/min/1.73 m^2^, CKD<15 chronic kidney disease with eGFR < 15 ml/min/1.73 m^2^, PD, peritoneal dialysis; HD, hemodialysis.

a Mean (SD),

b median (IQR), N/A, not applicable.

### Household expenditure and out-of-pocket expenditure

The total household expenditures (effective income) were similar in CKD15-60 compared to HD or PD in all schemes (Supplementary Table 2). Patient income and/or the total household expenditures were lower in UCS than CSMBS in all CKD groups (Supplementary Table 3).

**Figure 1:**
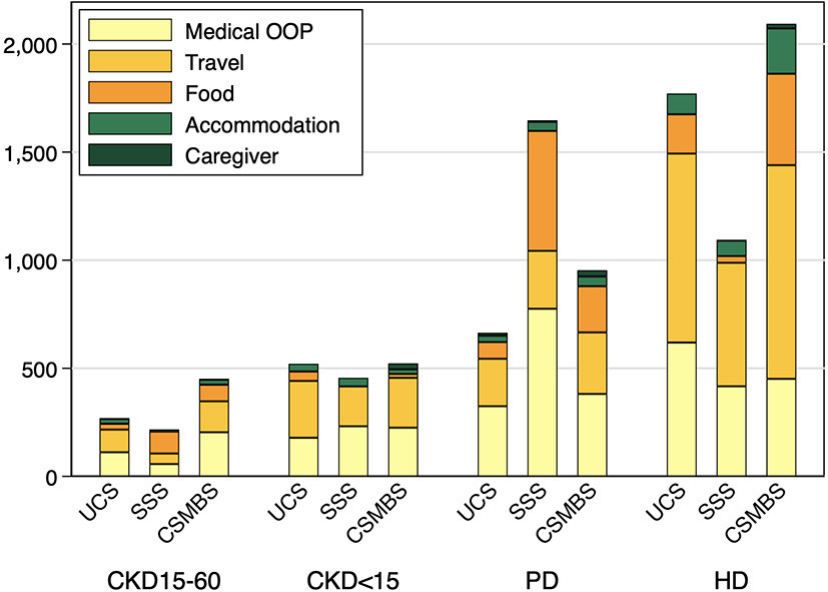
Breakdown of mean annual out-of-pocket cost by health insurance schemes and CKD groups. UCS, Universal Coverage Scheme; SSS, Social Security System; CSMBS, Civil Servant Monetary Benefit Scheme. CKD15-60 chronic kidney disease with eGFR 15–60 ml/min/1.73 m^2^, CKD<15 chronic kidney disease with eGFR < 15 ml/min/1.73 m^2^, PD, peritoneal dialysis; HD, hemodialysis.

The total OOPE in pre-dialysis CKD was comparable across all health schemes (Supplementary Table 3). Dialysis patients had higher total OOPE than pre-dialysis patients in all schemes, with HD having larger OOPE than PD in UCS and CSMBS. Under UCS, the total OOPE in HD was over two times higher than PD and nearly six times higher than CKD15-60. (Total OOPE (USD/year) for UCS: CKD15-60, 302 (205–400); CKD<15, 626 (311–941); PD, 759 (580–938); HD, 1,775 (1,262–2,288), *p* < 0.001). A similar trend was observed under CSMBS, but the OOPE was higher in CSMBS compared to UCS (Supplementary Tables 2, 3). Both medical and non-medical costs contributed to the marked increase in total OOPE in HD and PD patients. Travel cost was a major driver of OOPE in HD patients in all three schemes accounting for 44–49.3% of total OOPE (Figure 1, Supplementary Table 4). In contrast to other schemes, the OOPE under SSS was highest in PD, partly due to higher medical costs.

### Catastrophic health expenditure

CHE40 ranged from 0 to 11% in pre-dialysis CKD (Table 2, Supplementary Figure 2A). CHE40 was higher in dialysis patients compared to pre-dialysis CKD in all schemes. For UCS, CHE40 were: 8.5, 9.3, 19.5, and 50.0%, for CKD15-60, CKD<15, PD, and HD, respectively (*p* < 0.001). A similar pattern was seen in CSMBS, although the differences between PD (25%) and HD (39%) were less marked. By comparison, in SSS patients, CHE40 was higher in PD than HD.

**Table 2:** Proportion of catastrophic health expenditure (CHE40) and impoverishment by CKD groups.

	**Total (95%CI)**	**CKD15-60 (95%CI)**	**CKD<15 (95%CI)**	**PD (95%CI)**	**HD (95%CI)**	***P*-value**
**CHE40** ^a^						
**UCS**	19.6% (16.3–23.0)	8.5% (4.1–12.9)	9.3% (3.8–14.7)	19.5%^*^^$^ (13.8–25.2)	50.0%^*^^$^^#^ (39.9–60.1)	**< 0.001**
**SSS**	24.8% (16.7–32.9)	8.3% (−2.7–19.4)	0.0% (0.0–0.0)	54.5%^*^^$^ (25.1–84.0)	32.2%^*^^$^ (20.3–44.1)	**0.001**
**CSMBS**	19.5% (16.2–22.7)	10.9% (7.1–14.6)	5.6% (0.8–10.3)	24.6%^*^^$^ (13.8–35.4)	38.6%^*^^$^^#^ (31.2–46.0)	**< 0.001**
**Medical impoverishment**						
**UCS**						
Pre-out-of-pocket impoverishment^b^	16.1% (13.0–19.2)	18.3% (12.2–24.4)	11.1% (5.2–17.0)	15.7% (10.4–20.9)	19.1% (11.2–27.1)	0.348
Medical impoverishment^c^	12.1% (9.1–15.1)	8.0% (3.2–12.8)	3.1% (−0.4–6.6)	11.5%^$^ (6.5–16.6)	31.6%^*^^$^^#^ (21.1–42.0)	< 0.001
**SSS**						
Pre-out-of-pocket impoverishment^b^	5.5% (1.2–9.8)	0.0% (0.0–0.0)	0.0% (0.0–0.0)	9.1% (−7.9–26.1)	8.5% (1.4–15.6)	0.374
Medical impoverishment^c^	13.6% (7.0–20.2)	4.2% (−3.8–12.2)	0.0% (0.0–0.0)	0.0% (0.0–0.0)	24.1% (12.7–35.5)	**0.016**
**CSMBS**						
Pre-out-of-pocket impoverishment^b^	3.7% (2.1–5.2)	3.9% (1.5–6.2)	4.4% (0.2–8.7)	4.9% (−0.5–10.3)	2.4% (0.1–4.7)	0.668
Medical impoverishment^c^	7.6% (5.4–9.8)	4.8% (2.2–7.5)	4.7% (0.2–9.1)	6.9% (0.4–13.4)	13.6%^*^^$^^#^ (8.3–18.9)	**0.011**

UCS, Universal Coverage Scheme; SSS, Social Security System; CSMBS, Civil Servant Monetary Benefit Scheme; 95% CI, 95% Confidence Interval.

CKD15-60 chronic kidney disease with eGFR 15-60 ml/min/1.73 m^2^, CKD<15 chronic kidney disease with eGFR < 15 ml/min/1.73 m^2^, PD, peritoneal dialysis; HD, hemodialysis.

* P < 0.05 vs. CKD15-60,

$ P < 0.05 vs. CKD<15,

# P < 0.05 vs. PD.

a The percentage of households in which out-of-pocket payments for health care was at least 40% of household capacity to pay.

b The percentage of households in which total household expenditure was less than computed subsistence expenditure.

c The percentage of households in which total household expenditure after paying out-of-pocket for health was less than computed subsistence expenditure.

Bold values mean *P* value < 0.05.

**Figure 2:**
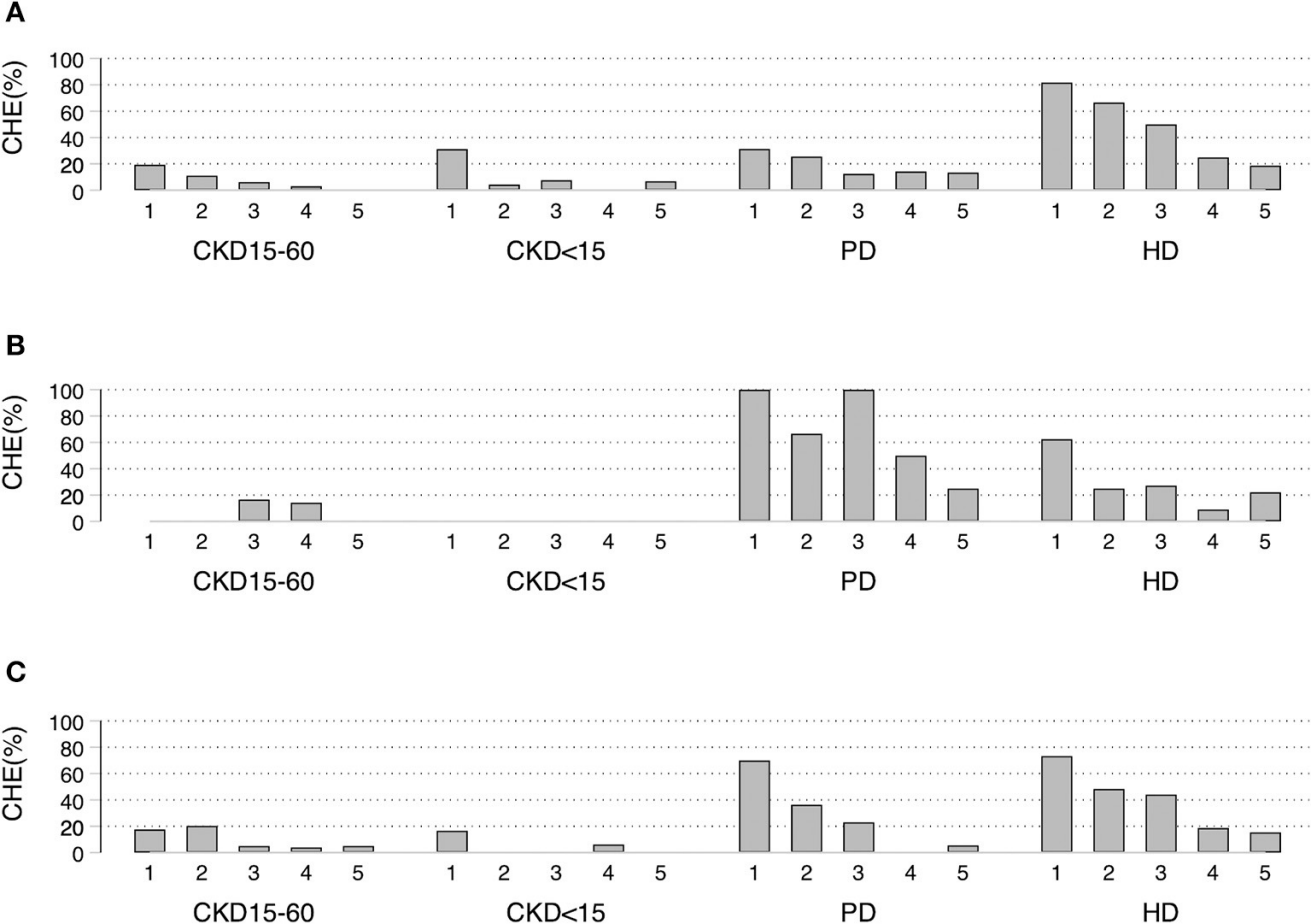
Socioeconomic status quintiles-specific proportion of Catastrophic Health Expenditure (CHE40) under different schemes. **(A)** UCS, **(B)** SSS, **(C)** CSMBS CHE40 defined as households in which out-of-pocket payments for health care was at least 40% of household capacity to pay. UCS, Universal Coverage Scheme; SSS, Social Security System; CSMBS, Civil Servant Monetary Benefit Scheme. CKD15-60 chronic kidney disease with eGFR 15-60 ml/min/1.73 m^2^, CKD<15 chronic kidney disease with eGFR < 15 ml/min/1.73 m^2^, PD, peritoneal dialysis; HD, hemodialysis.

CHE40 was higher in the lowest socioeconomic quintile, with more dialysis patients affected than pre-dialysis CKD. In the poorest quintile of UCS, the CHE40 were: 19, 32, 31, and 82% for CKD15-60, CKD<15, PD, and HD, respectively (*p* < 0.001). For the poorest quintile of CSMBS, about 70% of PD and HD patients had CHE40 compared to about 17% of pre-dialysis CKD (Figures 2A–C, Supplementary Tables 5A–C).

In the sensitivity analysis (Supplementary Tables 3A–D, 6), the results showed the same trend, but the proportions of CHE10 were higher. CHE10 under UCS were CKD15-60 15.7%, CKD<15 19.4%, PD, 40.5%, and HD 67.0% (*p* < 0.001).

### Medical impoverishment

The pre-out-of-pocket impoverishment (the total household expenditure below the computed subsistence expenditure) was higher in UCS (16%) compared to SSS (6%) and CSMBS (4%) (*p* < 0.001) (Table 2, Supplementary Figure 2B). Pre-out-of-pocket impoverishment was similar across CKD groups in UCS and CSMBS. Medical impoverishment was most common in all schemes in HD patients, being highest in UCS. The proportion with medical impoverishment under UCS were: 8.0, 3.1, 11.5, 31.6% for CKD15-60, CKD<15, PD, and HD, respectively (*p* < 0.001). Medical impoverishment in pre-dialysis CKD and PD were not different between UCS and CSMBS.

The proportion of medical impoverishment in the poorest quintile of patients was highest in HD in all schemes affecting 100, 55, and 50% of UCS, SSS, CSMBS, respectively (Supplementary Tables 7A–C, Supplementary Figures 3A–C). In the poorest quintile of UCS, medical impoverishment was also considerable in PD (67%) and pre-dialysis CKD (50%) patients. These values compare to 28% of PD and 12–20% of pre-dialysis CKD patients in the lowest quintile of CSMBS (Supplementary Tables 7A–C, Supplementary Figures 3A–C).

In the sensitivity analysis, HD patients still had the highest rate of impoverishment in all insurance schemes using the poverty line to define impoverishment (Supplementary Tables 3A–D, 6). For UCS, the medical impoverishment based on poverty line were CKD15-60 4.6%, CKD<15 3.7%, PD 3.8%, HD 20%.

### Factors associated with CHE

Compared with CKD15-60, PD and HD increased the adjusted risk of CHE40 by 3.3 and 8.8 folds, respectively (Table 3). After inclusion of the interaction between health schemes and CKD groups into the model, CHE40 risk in UCS in PD and HD increased by 3.5 and 16.3 folds, respectively. A similar pattern was seen for CSMBS, whereas in SSS, PD had a greater risk of CHE compared to HD. Other significant risk factors were older age, cardiovascular disease, absence of hypertension, and low numbers of household members.

**Table 3:** Multivariable analysis of health insurance schemes and CKD groups on CHE.

**Covariates (reference)**	**CHE40^a^**			**CHE10^b^**		
	**AOR^c^**	**95% CI**	***P*-value**	**AOR^c^**	**95% CI**	***P*-value**
**Health insurance schemes (UCS)**						
SSS	0.947	0.446–2.009	0.886	1.262	0.808–1.971	0.306
CSMBS	0.903	0.590–1.381	0.636	1.180	0.963–1.446	0.111
**CKD groups (CKD15-60)**						
CKD<15	0.709	0.371–1.355	0.298	1.525	1.017–2.285	**0.041**
PD	3.321	2.072–5.322	**< 0.001**	4.459	2.332–8.528	**< 0.001**
HD	8.828	5.295–14.718	**< 0.001**	11.084	8.073–15.218	**< 0.001**
**Interaction between health insurance schemes and CKD (UCS, CKD15-60)**						
UCS, CKD<15	1.188	0.691–2.042	0.534	1.324	0.860–2.039	0.202
UCS, PD	3.533	1.598–7.813	**0.002**	4.584	1.961–10.714	**< 0.001**
UCS, HD	16.280	8.173–32.430	**< 0.001**	14.390	8.671–23.883	**< 0.001**
SSS, CKD15-60	1.724	0.278–10.693	0.559	0.712	0.144–3.515	0.677
SSS, CKD<15	NA			2.595	1.267–5.316	**0.009**
SSS, PD	21.153	5.856–76.406	**< 0.001**	19.513	5.805–65.589	**< 0.001**
SSS, HD	8.301	3.073–22.428	**< 0.001**	12.286	6.507–23.198	**< 0.001**
CSMBS, CKD15-60	1.314	0.778–2.220	0.307	1.284	0.909–1.815	0.156
CSMBS, CKD<15	0.619	0.232–1.651	0.338	2.114	1.091–4.097	**0.027**
CSMBS, PD	4.946	2.387–10.247	**< 0.001**	4.577	1.884–11.120	**0.001**
CSMBS, HD	9.394	5.198–16.978	**< 0.001**	12.679	7.663–20.978	**< 0.001**

AOR, adjusted odds ratio; 95% CI, 95% Confidence Interval; NA, cannot be calculated due to small number of patients.

UCS, Universal Coverage Scheme; SSS, Social Security System; CSMBS, Civil Servant Monetary Benefit Scheme.

CKD15-60 chronic kidney disease with eGFR 15-60 ml/min/1.73 m^2^, CKD<15 chronic kidney disease with eGFR < 15 ml/min/1.73 m^2^, PD, peritoneal dialysis; HD hemodialysis.

a The percentage of households in which out-of-pocket payments for health care was at least 40% of household capacity to pay.

b The percentage of households in which out-of-pocket payments for health care was more than 10% of households' total consumption expenditure.

c Adjusted with age, sex, diabetes, hypertension, cardiovascular disease, dyslipidemia, annual patient income, number of household members.

Bold values mean *P* value < 0.05.

### The probability of CHE

The adjusted probability of CHE40 ranged from 5 to 12% for pre-dialysis CKD (Supplementary Table 8). Under UCS, the adjusted probability of CHE40 was higher (*p* < 0.05) in HD (52.7%) compared to PD (21.5%), CKD<15 (8.8 %), and CKD15-60 (7.6 %) (Supplementary Figure 4). CSMBS showed a similar trend, but the differences between HD (40.3%) and PD (27.2%) did not reach statistical significance (Supplementary Table 8, Supplementary Figure 4). The results for CHE10 were in a similar direction as the main findings (Table 3, Supplementary Table 8).

### The probability of CHE by geographic regions

We also analyzed the effect of different regions on the probability of CHE. We found that the adjusted prevalence of CHE40 was higher in the Central compared to the North, East and South regions, and the adjusted prevalence of CHE40 was higher in the Northeast compared to the North, and East regions. The adjusted prevalence of CHE10 also showed a higher prevalence of the Central region (Supplementary Table 9).

## Discussion

Despite universal coverage, there was substantial residual financial hardship in CKD patients, increasing from pre-dialysis to PD to HD. Under UCS and the “PD First Policy,” HD patients had the largest financial burden, whereas PD patients had a lower burden. Half of the HD patients had CHE, and 20% had medical impoverishment compared to 20 and 11% of PD patients. In the poorest UCS patients, medical impoverishment was almost 100% in HD and over 60% in PD. Non-medical costs especially traveling costs, were the main out-of-pocket expenditure in HD.

UCS reduced the burden of health care, especially among the poor (21, 22). In the Thai population, medical impoverishment (using the national poverty line) decreased from 2.3% in 1990 to 0.3% in 2015. Over the same period, CHE decreased from 7.1 to 2.1% (12), which is several folds lower than the global proportion of 12% (23). Previously, there have been no studies on the residual financial burden in CKD under universal coverage. Our data showed that CHE or medical impoverishment (defined by the poverty line) in pre-dialysis CKD was about 10 folds above the population average (21, 22). CKD patients have multiple co-morbidities. Travel costs to tertiary centers contribute to the out-of-pocket expenditure, whereas medical costs account for less than one-third of all out-of-pocket expenditure as health schemes cover most medication costs. Pre-dialysis CKD patients were better off than dialysis patients because their health status was generally better with less frequent hospital visits.

Since the initiation of dialysis coverage under the “PD First Policy,” the number of cases of kidney replacement in Thailand increased from 21,839 in 2007 to 164,191 in 2020, while PD increased from 5.5 to 21% of dialysis patients (7, 24). This massive increase was only achievable with the UCS program, as self-payment is too expensive for most patients (25). Nonetheless, our study shows that despite universal coverage, kidney failure still results in a substantial financial burden, especially in patients on HD.

Although data on the cost to the government for providing dialysis services in LMICs are available (3, 4), so far, very few studies have investigated the cost implications of CKD from the patients' perspective relative to their income. Without universal coverage, the burden of HD on patients in an LMIC is enormous. A recent study in HD patients from Kerala state, India, found that over 90% of households, who mainly did not have financial assistance, had CHE (26). Hemodialysis and medical costs were the main drivers of out-of-pocket expenditure in these privately funded patients (26). The lower CHE in our HD patients partly reflects the benefits of government coverage. The cost of HD for SSS or CSMBS patients or UCS patients with contraindications to PD is fully covered in government centers, but there may be extra co-payments in private centers. Co-payment for pre-approved HD, medications not listed in essential drug lists, and other health services, including vascular access formation at a non-registered hospital, accounted for higher medical out-of-pocket expenditure among HD patients under UCS than those under CSMBS (12, 27). With the dialysis cost being mostly covered, frequent traveling was a major out-of-pocket expenditure in HD under all health schemes consistent with other studies (26, 28).

The lower earnings of UCS patients increases the risk for CHE in the face of higher out-of-pocket expenditure incurred during HD, with the poorest suffering more from this excess burden. The higher cost of HD and the requirement of specialized centers and staff means that LMICS that has offered HD as an initial modality under universal coverage may have difficulty in achieving adequate dialysis coverage due to a lack of hemodialysis centers in remote areas (29). In addition, patients may skip dialysis sessions as they cannot pay the extra out-of-pocket costs in countries where hemodialysis coverage is only partial (30).

Few other studies have compared the financial burden across the spectrum from pre-dialysis CKD to PD and HD in the LMIC. Bello et al. showed that the percentage of monthly spending on health was 5-fold higher in HD than PD patients in a small study in South-African children (31). In our study, the odds of developing CHE under UCS were 2-fold higher for HD than for PD. The probability of CHE of PD under UCS is comparable to HD or PD under CSMBS despite lower income in the UCS group. The lower financial burden of PD compared to HD under UCS is consistent with the benefit of the “PD First Policy,” especially for the poor. PD is a home-based treatment with comparable outcomes to HD and requires less travel time (10). The lower need for health personnel and infrastructure allows greater access in remote areas and is more cost-effective than HD. In addition to lower traveling needs, the lower out-of-pocket expenditure for PD is dependent on the provision of free dialysate in the UCS scheme (9). The higher out-of-pocket expenditure and CHE rate for SSS may reflect incomplete reimbursement for PD in this scheme. In countries where peritoneal fluid cannot be imported cheaply, the cost-benefit of a “PD First” program may be altered (5). The higher prevalence of CHE in the Central region may reflect higher cost of living.

Our study has several strengths. This study is the first multicenter nationwide study to describe the residual financial burden of CKD patients under universal coverage and the “PD First Policy” to allow true insight into the economic impact on CKD households in Thailand. To our knowledge, we are among the first to evaluate the patient financial burden in a spectrum of CKD and dialysis patients using data obtained directly from patients in an LMIC.

There were several limitations in our study. Firstly, the cross-sectional design may not capture the fluctuation of expenditures throughout the year. Secondly, interview data may be subjected to recall bias. This study excluded the tiny proportion of UCS patients who used HD without approved indications and must cover the total treatment price for dialysis. Finally, our study contained relatively few SSS patients which may lead to bias in our data from this group.

This study provides data for policymakers in LMICs that should be useful in selecting the preferred dialysis modality for universal coverage (10, 30). Our study should warn policymakers of HD's considerable financial hardship. Without full knowledge of the hidden out-of-pocket expenditure, choosing HD could be catastrophic for many households in the long term. Financial distress in many dialysis patients should lead to strategies to support at-risk patients including more hemodialysis facilities in remote areas or transportation services for patients for whom HD is the only viable option (32). However, whether these options are feasible needs to be evaluated in the local context. Finally, it is important to consider that the dimension of the natural history of CKD is a continuous process (transitioning at different rates from one stage to the next) and so as the burden of disease, economic consequences and risk of CHE and medical impoverishment are also dependent on time that people have lived in the previous stage (and what they had already spent).

## Conclusion

Kidney failure patients had increased catastrophic health expenditure and medical impoverishment than pre-dialysis CKD. Under the “PD First” program for UCS, the financial hardship for patients on PD was considerably lower than HD, although the rates remained alarmingly high in the poor.

## Data availability statement

The raw data supporting the conclusions of this article will be made available by the authors, without undue reservation.

## Ethics statement

The studies involving human participants were reviewed and approved by Research Ethics Board at Central Research Ethics Committee (ID: COA-CREC 005/57). The patients/participants provided their written informed consent to participate in this study.

## Author contributions

PSangt, PTanv, PSangs, NC, and CKi were responsible for concept and design of the study. PSr and CKi were responsible for funding. PSangt, PK, SA, SB, PG, CKu, PL, KN, WP, SS, PTank, and PTar were responsible for patient evaluation and data collection. PSangt, SG, PTanv, PSangs, NC, and CKi were responsible for data analysis. PSangt, NC, and CKi were responsible for first draft of the manuscript. All authors were responsible for writing the final version of the manuscript and approving the final version.

## Funding

PSr was funded by HSRI grant ID 57-108. CKi was funded by HSRI grant ID 60-078. The study funders had no role in study design, data collection, analysis, interpretation or writing the report. NC and CKi had full access to all study data and had final responsibility for the decision to submit for publication.

## Conflict of interest

The authors declare that the research was conducted in the absence of any commercial or financial relationships that could be construed as a potential conflict of interest.

## Publisher's note

All claims expressed in this article are solely those of the authors and do not necessarily represent those of their affiliated organizations, or those of the publisher, the editors and the reviewers. Any product that may be evaluated in this article, or claim that may be made by its manufacturer, is not guaranteed or endorsed by the publisher.
