# Supplementary material for: The hidden financial catastrophe of chronic kidney disease under universal coverage and Thai “Peritoneal Dialysis First Policy”
Source: Front Public Health. 2022 Oct 13;10:965808. doi: 10.3389/fpubh.2022.965808 (PMC9606783; doi:10.3389/fpubh.2022.965808)
Supplement: Supplementary file 1 [file Table_1.DOCX]

**The hidden financial catastrophe of chronic kidney disease under universal coverage and Thai ‘Peritoneal Dialysis First Policy’**

Pornpen Sangthawan^1^, Pinkaew Klyprayong ^2^, Sarayut L. Geater^1^, Pimwara Tanvejsilp^3^ , Sirirat Anutrakulchai^4^, Sarinya Boongird^2^, Pongsathorn Gojaseni^5^, Charan Kuhiran^6^, Pichet Lorvinitnun^7^, Kajohnsak Noppakun^8^ , Watanyu Parapiboon^9^, Supinda Sirilak^10^, Pluemjit Tankee^11^, Puntapong Taruangsri^12^, Pasuree Sangsupawanich^13^ , Piyamitr Sritara^2^, Nathorn Chaiyakunapruk^14^*, Chagriya Kitiyakara* ^2^

**Supplementary materials**

**Supplementary Questionnaire.** Questionnaire for health economic data collection

**Supplementary Table S1.** Characteristics of Thailand’s main health insurance schemes^a^

**Supplementary Table S2.** Socioeconomic characteristics and out-of-pocket expenditures by CKD groups

**Supplementary Table S3.** Socioeconomic, out-of-pocket (OOP) expenditures, Catastrophic Health Expenditure (CHE) and impoverishment by health insurance schemes in CKD groups (**Supplementary** **Table S3A-D**)

**Supplementary Table S4.** Out-of-pocket expenditure as the percentage of total out-of-pocket expenditures for health

**Supplementary Table S5.** Socioeconomic status quintiles-specific proportion of Catastrophic Health Expenditure (CHE) (**Supplementary** **Table S5A-C**)

**Supplementary Table S6.** Proportion of Catastrophic Health Expenditure (CHE10) and impoverishment using poverty line by CKD groups

**Supplementary Table S7.** Socioeconomic status quintiles-specific proportion of pre-out-of-pocket and impoverishment (**Supplementary** **Table S7A-C**)

**Supplementary Table S8.** Multivariable adjusted probability of Catastrophic Health Expenditure (CHE)

**Supplementary Table S9** Probability of incurring Catastrophic Health Expenditure (CHE) by regions from the modeling

**Supplementary Figure S1.** Flow of study

**Supplementary Figure S2.** Proportion of Catastrophic Health Expenditure (CHE40) ^a^ and impoverishment ^b,c^ according to CKD groups and health insurance schemes

**Supplementary Figure S3.** Socioeconomic status quintiles-specific proportion of pre-out-of-pocket (pre-OOP)^a^ and medical impoverishment^b^

**Supplementary Figure S4.** Probability of Catastrophic Health Expenditure (CHE40)^a^ by Health insurance schemes and CKD groups

**Supplementary Questionnaire** Questionnaire for health economic data collection

**Table S1** Characteristics of Thailand’s main health insurance schemes^a^

| **Health insurance scheme** | **UCS** | **SSS** | **CSMBS** |
| --- | --- | --- | --- |
| Population coverage | the rest of Thai people | private sector employees, excluding dependants | government employees and dependants |
| Percentage coverage | 75 | 16 | 9 |
| Source of revenue | General tax | Tripartite contribution, equally shared by employer, employee and government | General tax,  Non-contributary scheme |
| Mode of provider payment | Capitation for outpatient and global budget plus Diagnostic Related Group (DRG) for inpatient | Inclusive capitation for both outpatient and inpatient plus additional adjusted payments for accident and emergency and high-cost care | Fee for service, direct disbursement to mostly public providers and DRG for inpatient treatment |
| Access to service | Registered contractors, the network of public hospitals (contracting unit for primary care) | Registered public and private contraction | Free choice of public provider |
| Dialysis cost | Free for PD as first modality,  Reimbursable for HD if contraindicated to PD | Fixed fee for HD and PD  Monthly extra-payment for PD  And HD (some private providers) | Free for HD, PD  Extra-payment for some medical supply |
| Medicines | Free for medicines under essential drugs list  Erythropoietin through capitations | Free for medicines under essential drugs list  Erythropoietin through capitations | Free for medicines under essential drugs list  Erythropoietin as needed |
| Surgical procedures associated with dialysis (vascular access, Tenchkoff catheter insertion) | Fixed fee | Fixed fee | Free for public provider |

UCS, Universal Coverage Scheme. SSS, Social Security System. CSMBS, Civil Servant Monetary Benefit Scheme. PD Peritoneal dialysis, HD Hemodialysis

^a^ Data adapted from Tangcharoensathien V, Tisayaticom K, Suphanchaimat R, Vongmongkol V, Viriyathorn S, Limwattananon S. Financial risk protection of Thailand's universal health coverage: results from series of national household surveys between 1996 and 2015. *Int J Equity Health*. Sep 21 2020;19(1):163. doi:10.1186/s12939-020-01273-6

**Table S2** Socioeconomic characteristics and out-of-pocket expenditures by CKD groups

| **Characteristics** | **Total** | **CKD15-60** | **CKD<15** | **PD** | **HD** | **P-value** |
| --- | --- | --- | --- | --- | --- | --- |
| **Socioeconomic characteristics** |  |  |  |  |  |  |
| **UCS** (%) | 540 (100) | 153 (28) | 108 (20) | 185 (34) | 94 (18) |  |
| Patient income ^a^ | 1549  (1221-1876) | 1612  (1092-2132) | 2052  (930-3174) | 1259  (874-1645) | 1437  (670-2204) | 0.399 |
| Total household expenditures ^a^ | 6174  (5767-6580) | 5570  (4940-6200) | 7193*  (6013-8372) | 6414  (5735-7093) | 5513  (4670-6356) | **0.023** |
| **SSS** (%) | 109 (100) | 24 (22) | 15 (14) | 11 (10) | 59 (54) |  |
| Patient income ^a^ | 4170  (3284-5055) | 7220  (4930-9509) | 4304  (2322-6286) | 3029*  (326-5731) | 3108*  (2,091-4124) | **0.003** |
| Total household expenditures ^a^ | 7077  (6192-7961) | 7666  (6252-9080) | 6951  (5374-8528) | 7777  (5529-10026) | 6738  (5314-8163) | 0.821 |
| **CSMBS** (%) | 575 (100) | 258 (44) | 90 (16) | 61 (11) | 166 (29) |  |
| Patient income ^a^ | 6457  (5731-7183) | 5891  (5152-6630) | 5627  (4190-7064) | 9436*  (5295-13576) | 6693*  (5266-8120) | **0.032** |
| Total household expenditures ^a^ | 9250  (8681-9819) | 8682  (7905-9458) | 8714  (7338-10090) | 11070  (8650-13490) | 9756  (8728-10784) | 0.063 |
| **Out-of-pocket expenditures** |  |  |  |  |  |  |
| **UCS** (%) | 540 (100) | 153 (28) | 108 (20) | 185 (34) | 94 (18) |  |
| Out-of-pocket for medical expenditures ^a^ | 286  (204-369) | 112  (70-153) | 179  (72-285) | 325*^$^  (245-404) | 619^#^  (200-1038) | **0.001** |
| Out-of-pocket for non-medical expenditures ^a^ | 494  (396-591) | 191  (110-271) | 447*  (170-725) | 434*^$^  (283-586) | 1156*^$^ ^#^  (863-1449) | **< 0.001** |
| Total out-of-pocket expenditures ^a^ | 780  (645-914) | 302  (205-400) | 626*  (311-941) | 759*^$^  (580-938) | 1775*^$ #^  (1262-2288) | **< 0.001** |
| **SSS** (%) | 109 (100) | 24 (22) | 15 (14) | 11 (10) | 59 (54) |  |
| Out-of-pocket for medical expenditures ^a^ | 348  (192-504) | 57  (12-103) | 232  (78-386) | 775  (396-1154) | 416  (148-685) | 0.086 |
| Out-of-pocket for non-medical expenditures ^a^ | 685  (434-936) | 527  (-215-1269) | 224  (39-409) | 1,057  (-68-2182) | 797  (516-1078) | 0.341 |
| Total out-of-pocket expenditures ^a^ | 1033  (737-1329) | 584  (-158-1327) | 456  (206-706) | 1832  (580-3084) | 1213  (842-1585) | 0.054 |
| **CSMBS** (%) | 575 (100) | 258 (44) | 90 (16) | 61 (11) | 166 (29) |  |
| Out-of-pocket for medical expenditures ^a^ | 297  (243-352) | 204  (121-287) | 225  (142-308) | 381*^$^  (286-477) | 451*^$^ ^#^  (329-574) | **0.001** |
| Out-of-pocket for non-medical expenditures ^a^ | 834  (670-998) | 368  (239-497) | 383  (247-519) | 863*  (399-1328) | 1,790*^$ #^  (1323-2258) | **< 0.001** |
| Total out-of-pocket expenditures ^a^ | 1131  (951-1312) | 572  (415-730) | 608  (435-781) | 1245*^$^  (751-1739) | 2242*^$ #^  (1744-2740) | **< 0.001** |

UCS, Universal Coverage Scheme. SSS, Social Security System. CSMBS, Civil Servant Monetary Benefit Scheme

CKD15-60 chronic kidney disease with eGFR 15-60 ml/min/1.73m^2^, CKD<15 chronic kidney disease with eGFR<15 ml/min/1.73m^2^, PD peritoneal dialysis, HD hemodialysis

^a^ annual, mean (95% CI) (USD, 2021)

*P-value <0.05 vs CKD15-60, ^$^ P-value <0.05 vs CKD<15, ^#^ P-value <0.05 vs PD

**Table S3** Socioeconomic, out-of-pocket (OOP) expenditures, Catastrophic Health Expenditure (CHE) and impoverishment by health insurance schemes in CKD groups

**Table S3A** Socioeconomic, OOP expenditures, CHE and impoverishment by health insurance schemes in CKD15-60

|  | **Total** | **UCS** | **SSS** | **CSMBS** | **P-value** |
| --- | --- | --- | --- | --- | --- |
| N (%) | 435 (100.0) | 153(35.2) | 24(5.5) | 258(59.3) |  |
| **Socioeconomic characteristics** |  |  |  |  |  |
| Patient income ^a^ | 4460  (5633) | 1612  (3282) | 7220*  (5723) | 5891*  (6057) | **< 0.001** |
| Total household expenditure ^a^ | 7531  (5684) | 5570  (3977) | 7666*  (3535) | 8682*  (6361) | **< 0.001** |
| **Out-of-pocket expenditure** |  |  |  |  |  |
| OOP for medical expenditure ^a^ | 163 (550) | 112 (264) | 57 (113) | 204 (681) | 0.160 |
| OOP for non-medical expenditure ^a^ | 315 (972) | 191 (510) | 527 (1854) | 368 (1058) | 0.109 |
| Total OOP expenditures ^a^ | 478 (1148) | 302 (616) | 584 (1856) | 572 (1290) | 0.062 |
| **CHE and impoverishment** |  |  |  |  |  |
| CHE40 ^b^, (95% CI) | 9.9%  (7.1-12.7) | 8.5%  (4.1-12.9) | 8.3%  (-2.7-19.4) | 10.9%  (7.1-14.6) | 0.799 |
| Pre-OOP impoverishment ^c^, (95% CI) | 8.7%  (6.1-11.4) | 18.3%  (12.2-24.4) | 0.0%*  (0.0-0.0) | 3.9%*  (1.5-6.2) | **< 0.001** |
| Medical impoverishment ^d^, (95% CI) | 5.8 (3.5-8.1) | 8.0 (3.2-12.8) | 4.2 (-3.8-12.2) | 4.8 (2.2-7.5) | 0.416 |
| **CHE and impoverishment** |  |  |  |  |  |
| CHE10 ^e^, (95% CI) | 17.7%  (14.1-21.3) | 15.7%  (9.9-21.4) | 8.3%  (-2.7-19.4) | 19.8%  (14.9-24.6) | 0.332 |
| Pre-OOP impoverishment ^f^, (95% CI) | 0.9%  (0.0-1.8) | 1.3%  (-0.5-3.1) | 0.0%  (0.0-0.0) | 0.8%  (-0.3-1.8) | 0.706 |
| Medical impoverishment ^g^, (95% CI) | 3.2%  (1.6-4.9) | 4.6%  (1.3-7.9) | 4.2%  (-3.8-12.2) | 2.3%  (0.5-4.2) | 0.341 |

CKD15-60 chronic kidney disease with eGFR 15-60 ml/min/1.73m^2^, UCS, Universal Coverage Scheme. SSS, Social Security System, CSMBS, Civil Servant Monetary Benefit Scheme

^a^ annual, mean (SD) (USD, 2021),

^b^ The percentage of households in which out-of-pocket payments for health care was at least 40% of household capacity to pay

^c^ The percentage of households in which total household expenditure was less than computed subsistence expenditure

^d^ The percentage of households in which total household expenditure after paying OOP for health, was less than computed subsistence expenditure

^e^:The percentage of households in which out-of-pocket payments for health care was more than 10 % of households’ total consumption expenditure

^f^:The percentage of households in which total household expenditure was less than poverty line

^g^:The percentage of households in which total household expenditure after paying OOP for health, was less than poverty line

*P-value <0.05 vs UCS, ^$^ P-value <0.05 vs SSS

**Table S3B** Socioeconomic, OOP expenditures, CHE and impoverishment by health insurance schemes in CKD< 15

|  | **Total** | **UCS** | **SSS** | **CSMBS** | **P-value** |
| --- | --- | --- | --- | --- | --- |
| N (%) | 213 (100.0) | 108 (50.7) | 15 (7.0) | 90 (42.3) |  |
| **Socioeconomic characteristics** |  |  |  |  |  |
| Patient income ^a^ | 3721 (6494) | 2052 (5949) | 4304 (3916) | 5627*(6955) | **< 0.001** |
| Total household expenditure ^a^ | 7818 (6294) | 7193 (6255) | 6951 (3116) | 8714 (6662) | 0.205 |
| **Out-of-pocket expenditure** |  |  |  |  |  |
| OOP for medical expenditure ^a^ | 202 (484) | 179 (563) | 232 (304) | 225 (401) | 0.776 |
| OOP for non-medical expenditure ^a^ | 405 (1134) | 447 (1471) | 224 (366) | 383 (660) | 0.755 |
| Total OOP expenditures ^a^ | 606 (1310) | 626 (1669) | 456 (494) | 608 (837) | 0.895 |
| **CHE and impoverishment** |  |  |  |  |  |
| CHE40 ^b^, (95% CI) | 7.0%  (3.6-10.5) | 9.3%  (3.8-14.7) | 0.0%  (0.0-0.0) | 5.6%  (0.8-10.3) | 0.479 |
| Pre-OOP impoverishment ^c^, (95% CI) | 7.5%  (4.0-11.1) | 11.1%  (5.2-17.0) | 0.0%  (0.0-0.0) | 4.4%  (0.2-8.7) | 0.152 |
| Medical impoverishment ^d^, (95% CI) | 3.6%  (1.0-6.1) | 3.1%  (-0.4-6.6) | 0.0%  (0.0-0.0) | 4.7%  (0.2-9.1) | 0.834 |
| **CHE and impoverishment** |  |  |  |  |  |
| CHE10 ^e^, (95% CI) | 23.95%  (18.2-29.7) | 19.4%  (12.0-26.9) | 26.7%  (4.3-49.0) | 28.9%  (19.5-38.3) | 0.296 |
| Pre-OOP impoverishment ^f^, (95% CI) | 0.9%  (-0.4-2.2) | 0.9%  (-0.9-2.7) | 0.0%  (0.0-0.0) | 1.1%  (-1.1-3.3) | 1.000 |
| Medical impoverishment ^g^, (95% CI) | 1.9%  (0.1-3.7) | 3.7%  (0.1-7.3) | 0.0%  (0.0-0.0) | 0.0%  (0.0-0.0) | 0.178 |

CKD<15 chronic kidney disease with eGFR<15 ml/min/1.73m^2^, UCS, Universal Coverage Scheme. SSS, Social Security System, CSMBS, Civil Servant Monetary Benefit Scheme

^a^ annual, mean (SD) (USD, 2021),

^b^ The percentage of households in which out-of-pocket payments for health care was at least 40% of household capacity to pay

^c^ The percentage of households in which total household expenditure was less than computed subsistence expenditure

^d^ The percentage of households in which total household expenditure after paying OOP for health, was less than computed subsistence expenditure

^e^:The percentage of households in which out-of-pocket payments for health care was more than 10 % of households’ total consumption expenditure

^f^:The percentage of households in which total household expenditure was less than poverty line

^g^:The percentage of households in which total household expenditure after paying OOP for health, was less than poverty line; *P-value <0.05 vs UCS, ^$^ P-value <0.05 vs SSS

**Table S3C** Socioeconomic, OOP expenditures, CHE and impoverishment by health insurance schemes in PD

|  | **Total** | **UCS** | **SSS** | **CSMBS** | **P-value** |
| --- | --- | --- | --- | --- | --- |
| N (%) | 257 (100.0) | 185 (72.0) | 11 (4.3) | 61 (23.7) |  |
| **Socioeconomic characteristics** |  |  |  |  |  |
| Patient income ^a^ | 3276 (9041) | 1259 (2674) | 3029 (4573) | 9436*^$^ (16499) | **< 0.001** |
| Total household expenditure ^a^ | 7577 (6497) | 6414 (4712) | 7777 (3805) | 11070* (9643) | **< 0.001** |
| **Out-of-pocket expenditure** |  |  |  |  |  |
| OOP for medical expenditure ^a^ | 357 (525) | 325 (549) | 775* (642) | 381*^$^ (379) | **0.019** |
| OOP for non-medical expenditure ^a^ | 563 (1336) | 434 (1054) | 1057 (1903) | 863*(1850) | **0.042** |
| Total OOP expenditure ^a^ | 920 (1508) | 759 (1243) | 1832 (2119) | 1245*(1968) | **0.011** |
| **CHE and impoverishment** |  |  |  |  |  |
| CHE40 ^b^, (95% CI) | 22.2%  (17.1-27.3) | 19.5%  (13.8-25.2) | 54.5%*  (25.1-84.0) | 24.6%  (13.8-35.4) | **0.027** |
| Pre-OOP impoverishment ^c^, (95% CI) | 12.8% (8.8-16.9) | 15.7% (10.4-20.9) | 9.1% (-7.9-26.1) | 4.9% (-0.5-10.3) | 0.074 |
| Medical impoverishment ^d^, (95% CI) | 9.8% (5.9-13.7) | 11.5% (6.5-16.6) | 0.0% (0.0-0.0) | 6.9% (0.4-13.4) | 0.531 |
| **CHE and impoverishment** |  |  |  |  |  |
| CHE10 ^e^, (95% CI) | 42.4%  (36.4-48.5) | 40.5%  (33.5-47.6) | 72.7%  (46.4-99.0) | 42.6%  (30.2-55.0) | 0.126 |
| Pre-OOP impoverishment ^f^, (95% CI) | 1.6 %  (0.0-3.1) | 2.2%  (0.1-4.3) | 0.0%  (0.0-0.0) | 0.0%  (0.0-0.0) | 0.643 |
| Medical impoverishment ^g^, (95% CI) | 3.5 % (1.3-5.7) | 3.8% (1.0-6.5) | 0.0% (0.0-0.0) | 3.3% (-1.2-7.7) | 1.000 |

PD Peritoneal dialysis, UCS, Universal Coverage Scheme. SSS, Social Security System, CSMBS, Civil Servant Monetary Benefit Scheme

^a^ annual, mean (SD) (USD, 2021),

^b^ The percentage of households in which out-of-pocket payments for health care was at least 40% of household capacity to pay

^c^ The percentage of households in which total household expenditure was less than computed subsistence expenditure

^d^ The percentage of households in which total household expenditure after paying OOP for health, was less than computed subsistence expenditure

^e^:The percentage of households in which out-of-pocket payments for health care was more than 10 % of households’ total consumption expenditure

^f^:The percentage of households in which total household expenditure was less than poverty line

^g^:The percentage of households in which total household expenditure after paying OOP for health, was less than poverty line

*P-value <0.05 vs UCS, ^$^ P-value <0.05 vs SSS

**Table S3D** Socioeconomic, OOP expenditures, CHE and impoverishment by health insurance schemes in HD

|  | **Total** | **UCS** | **SSS** | **CSMBS** | **P-value** |
| --- | --- | --- | --- | --- | --- |
| N (%) | 319 (100.0) | 94 (29.5) | 59 (18.5) | 166 (52.0) |  |
| **Socioeconomic characteristics** |  |  |  |  |  |
| Patient income ^a^ | 4481  (7641) | 1437  (3793) | 3108*  (3984) | 6693*^$^  (9379) | **< 0.001** |
| Total household expenditure ^a^ | 7948  (6182) | 5513  (4172) | 6738  (5582) | 9756*^$^  (6760) | **< 0.001** |
| **Out-of-pocket expenditure** |  |  |  |  |  |
| OOP for medical expenditure ^a^ | 494 (1342) | 619 (2072) | 416 (1052) | 451 (805) | 0.555 |
| OOP for non-medical expenditure ^a^ | 1420  (2429) | 1156  (1450) | 797  (1101) | 1790*^$^  (3073) | **0.012** |
| Total OOP expenditures ^a^ | 1914  (2825) | 1775  (2539) | 1213  (1455) | 2242*^$^  (3273) | **0.047** |
| **CHE and impoverishment** |  |  |  |  |  |
| CHE40 ^b^, (95% CI) | 40.8%  (35.4-46.1) | 50.0%  (39.9-60.1) | 32.2%  (20.3-44.1) | 38.6%  (31.2-46.0) | 0.070 |
| Pre-OOP impoverishment ^c^, (95% CI) | 8.5%  (5.4-11.5) | 19.1%  (11.2-27.1) | 8.5%  (1.4-15.6) | 2.4%*  (0.1-4.7) | **< 0.001** |
| Medical impoverishment ^d^, (95% CI) | 20.2%  (15.6-24.8) | 31.6%  (21.1-42.0) | 24.1%  (12.7-35.5) | 13.6%*  (8.3-18.9) | **0.004** |
| **CHE and impoverishment** |  |  |  |  |  |
| CHE10 ^e^, (95% CI) | 66.1  (61.0-71.3) | 67.0%  (57.5-76.5) | 62.7  (50.4-75.1) | 66.9  (59.7-74.0) | 0.822 |
| Pre-OOP impoverishment ^f^, (95% CI) | 1.3 %  (0.0-2.5) | 3.2%  (-0.4-6.7) | 1.7%  (-1.6-5.0) | 0.0 %  (0.0-0.0) | 0.065 |
| Medical impoverishment ^g^, (95% CI) | 11.0%  (7.5-14.4) | 20.2%  (12.1-28.3) | 10.2%  (2.5-17.9) | 6.0%*  (2.4-9.6) | **0.003** |

HD hemodialysis, UCS, Universal Coverage Scheme. SSS, Social Security System, CSMBS, Civil Servant Monetary Benefit Scheme

^a^ annual, mean (SD) (USD, 2021)

^b^ The percentage of households in which out-of-pocket payments for health care was at least 40% of household capacity to pay

^c^ The percentage of households in which total household expenditure was less than computed subsistence expenditure

^d^ The percentage of households in which total household expenditure after paying OOP for health, was less than computed subsistence expenditure

^e^:The percentage of households in which out-of-pocket payments for health care was more than 10 % of households’ total consumption expenditure

^f^:The percentage of households in which total household expenditure was less than poverty line

^g^:The percentage of households in which total household expenditure after paying OOP for health, was less than poverty line

*P-value <0.05 vs UCS, ^$^ P-value <0.05 vs SSS

**Table S4** Out-of-pocket expenditure as the percentage of total out-of-pocket expenditures for health

| **CKD groups** | **CKD15-60(%)** | | | **CKD<15(%)** | | | **PD(%)** | | | **HD(%)** | | |
| --- | --- | --- | --- | --- | --- | --- | --- | --- | --- | --- | --- | --- |
| **Health insurance schemes** | **UCS** | **SSS** | **CSMBS** | **UCS** | **SSS** | **CSMBS** | **UCS** | **SSS** | **CSMBS** | **UCS** | **SSS** | **CSMBS** |
| **Out-of-pocket for medical costs** | 36.9 | 9.8 | 35.7 | 28.5 | 50.8 | 37.0 | 42.8 | 42.3 | 30.6 | 34.8 | 34.4 | 20.1 |
| Out-of-pocket for medical costs  at OPD study hospital | 11.3 | 0.0 | 7.9 | 10.1 | 28.6 | 13.4 | 30.7 | 20.7 | 19.4 | 9.8 | 12.1 | 7.3 |
| Out-of-pocket for medical costs  at OPD other hospital | 2.7 | 0.0 | 8.2 | 1.4 | 6.1 | 4.2 | 0.7 | 5.4 | 1.7 | 23.3 | 12.3 | 3.6 |
| Out-of-pocket for medical costs  at IPD | 11.7 | 0.0 | 8.8 | 13.9 | 11.5 | 6.7 | 3.4 | 3.7 | 1.0 | 0.6 | 7.8 | 4.5 |
| Out-of-pocket for medical costs outside  Hospital | 11.2 | 9.8 | 10.8 | 3.2 | 4.6 | 12.7 | 8.0 | 12.5 | 8.5 | 1.1 | 2.2 | 4.7 |
| **Out-of-pocket for non-medical costs** | 63.1 | 90.2 | 64.3 | 71.5 | 49.2 | 63.0 | 57.2 | 57.7 | 69.4 | 65.2 | 65.6 | 79.9 |
| Food cost at OPD study hospital | 4.7 | 1.7 | 2.4 | 4.1 | 3.4 | 2.6 | 2.7 | 2.2 | 2.7 | 2.4 | 3.3 | 7.7 |
| Food cost at OPD other hospital | 0.3 | 0.0 | 0.2 | 0.3 | 0.4 | 0.2 | 0.2 | 0.1 | 0.0 | 2.7 | 2.3 | 1.6 |
| Food cost at IPD | 2.5 | 0.0 | 1.5 | 1.3 | 5.0 | 0.7 | 1.0 | 0.0 | 1.0 | 0.4 | 0.2 | 0.2 |
| Travel cost at OPD study hospital | 25.7 | 7.5 | 22.3 | 33.7 | 18.3 | 32.0 | 22.7 | 7.7 | 21.8 | 26.7 | 28.5 | 32.1 |
| Travel cost at OPD other hospital | 4.7 | 0.2 | 1.8 | 1.2 | 20.2 | 3.5 | 4.3 | 6.9 | 0.5 | 21.3 | 18.0 | 11.5 |
| Travel cost at IPD | 4.2 | 0.6 | 0.9 | 7.1 | 1.9 | 2.4 | 2.0 | 0.0 | 0.6 | 1.3 | 0.6 | 0.4 |
| Accommodation cost OPD study hospital | 0.2 | 0.0 | 0.7 | 0.0 | 0.0 | 4.5 | 1.7 | 0.0 | 2.3 | 0.0 | 0.0 | 0.9 |
| Accommodation cost OPD other hospital | 0.0 | 0.0 | 0.0 | 0.0 | 0.0 | 0.0 | 0.0 | 0.4 | 0.0 | 0.0 | 0.2 | 0.0 |
| House improvement | 11.9 | 62.8 | 21.1 | 16.8 | 0.0 | 14.1 | 12.5 | 10.1 | 23.4 | 0.2 | 10.0 | 6.6 |
| Formal caregiver | 8.9 | 17.4 | 13.4 | 6.9 | 0.0 | 3.0 | 10.1 | 30.3 | 17.1 | 10.2 | 2.5 | 18.9 |

UCS, Universal Coverage Scheme. SSS, Social Security System. CSMBS, Civil Servant Monetary Benefit Scheme

OPD outpatient department, IPD inpatient department

CKD15-60 chronic kidney disease with eGFR 15-60 ml/min/1.73m^2^, CKD<15 chronic kidney disease with eGFR<15 ml/min/1.73m^2^, PD peritoneal dialysis, HD hemodialysis

**Table S5** Socioeconomic status quintiles-specific proportion of Catastrophic Health Expenditure (CHE)

**Table S5A** Socioeconomic status quintiles-specific proportion of CHE in UCS

| **Quintiles of socioeconomic status** | **Total**  (95% CI) | **1**  (95% CI) | **2**  (95% CI) | **3**  (95% CI) | **4**  (95% CI) | **5**  (95% CI) | **P-value** |
| --- | --- | --- | --- | --- | --- | --- | --- |
| **CKD15-60** (N=153) |  |  |  |  |  |  |  |
| CHE40 ^a^ | 8.5%  (4.1-12.9) | 19.4%  (6.5-32.4) | 11.1%  (-0.7; 23.0) | 6.2%  (-2.1-14.6) | 3.1%  (-2.9-9.2) | 0.0%  (0.0-0.0) | 0.051 |
| CHE10 ^b^ | 15.7%  (9.9-21.4) | 33.3%  (17.9-48.7) | 22.2%  (6.5-37.9) | 12.5%  (1.0-24.0) | 6.2%  (-2.1-14.6) | 0.0%  (0.0-0.0) | **0.002** |
| **CKD<15** (N=108) |  |  |  |  |  |  |  |
| CHE40 ^a^ | 9.3%  (3.8-14.7) | 31.2%  (8.5-54.0) | 4.3%  (-4.0; 12.7) | 7.7%  (-2.6-17.9) | 0.0%  (0.0-0.0) | 6.9%  (-2.3-16.1) | 0.047 |
| CHE10 ^b^ | 19.4%  (12.0-26.9) | 31.2%  (8.5-54.0) | 17.4%  (1.9-32.9) | 23.1%  (6.9-39.3) | 0.0%  (0.0-0.0) | 20.7%  (5.9-35.4) | 0.230 |
| **PD** (N=185) |  |  |  |  |  |  |  |
| CHE40 ^a^ | 19.5%  (13.8-25.2) | 31.4%  (16.0-46.8) | 25.6%  (11.9-39.3) | 12.5%  (1.0-24.0) | 14.3%  (3.7-24.9) | 13.5%  (2.5-24.5) | 0.176 |
| CHE10 ^b^ | 40.5%  (33.5-47.6) | 51.4%  (34.9-68.0) | 41.0%  (25.6-56.5) | 28.1%  (12.5-43.7) | 45.2%  (30.2-60.3) | 35.1%  (19.8-50.5) | 0.336 |
| **HD** (N=94) |  |  |  |  |  |  |  |
| CHE40 ^a^ | 50.0%  (39.9-60.1) | 81.8%  (65.7-97.9) | 66.7%  (44.9-88.4) | 50.0%  (26.9-73.1) | 25.0%  (6.0-44.0) | 18.8%  (-0.4-37.9) | **<0.001** |
| CHE10 ^b^ | 67.0%  (57.5-76.5) | 90.9%  (78.9-102.9) | 72.2%  (51.5-92.9) | 61.1%  (38.6-83.6) | 60.0%  (38.5-81.5) | 43.8%  (19.4-68.1) | **0.024** |

UCS, Universal Coverage Scheme

CKD15-60 chronic kidney disease with eGFR 15-60 ml/min/1.73m^2^, CKD<15 chronic kidney disease with eGFR<15 ml/min/1.73m^2^, PD peritoneal dialysis, HD hemodialysis

^a^:The percentage of households in which out-of-pocket payments for health care was at least 40% of household capacity to pay

^b^:The percentage of households in which out-of-pocket payments for health care was more than 10 % of households’ total consumption expenditure

**Table S5B** Socioeconomic status quintiles-specific proportion of CHE in SSS

| **Quintiles of socioeconomic**  **status** | **Total**  (95% CI) | **1**  (95% CI) | **2**  (95% CI) | **3**  (95% CI) | **4**  (95% CI) | **5**  (95% CI) | **P-value** |
| --- | --- | --- | --- | --- | --- | --- | --- |
| **CKD15-60** (N=24) |  |  |  |  |  |  |  |
| CHE40 ^a^ | 8.3%  (-2.7-19.4) | 0.0%  (0.0- 0.0) | 0.0%  (0.0-0.0) | 16.7%  (-13.2-46.5) | 14.3%  (-11.6-40.2) | 0.0%  (0.0-0.0) | 1.000 |
| CHE10 ^b^ | 8.3%  (-2.7-19.4) | 0.0%  (0.0-0.0) | 0.0%  (0.0-0.0) | 16.7%  (-13.2- 46.5) | 14.3%  (-11.6-40.2) | 0.0%  (0.0-0.0) | 1.000 |
| **CKD<15** (N=15) |  |  |  |  |  |  |  |
| CHE40 ^a^ | 0.0%  (0.0-0.0) | 0.0%  (0.0-0.0) | 0.0%  (0.0-0.0) | 0.0%  (0.0-0.0) | 0.0%  (0.0-0.0) | 0.0%  (0.0-0.0) | - |
| CHE10 ^b^ | 26.7%  (4.3-49.0) | 0.0%  (0.0-0.0) | 50.0%  (1.0-99.0) | 25.0%  (-17.4-67.4) | 0.0%  (0.0-0.0) | 33.3%  (-20.0-86.7) | 0.859 |
| **PD** (N=11) |  |  |  |  |  |  |  |
| CHE40 ^a^ | 54.5%  (25.1-84.0) | 100.0%  (100.0-100.0) | 66.7%  (13.3-120.0) | 100.0%  (100.0-100.0) | 50.0%  (-19.31-19.3) | 25.0%  (-17.4-67.4) | 0.766 |
| CHE10 ^b^ | 72.7%  (46.4-99.0) | 100.0%  (100.0-100.0) | 100.0%  (100.0-100.0) | 100.0%  (100.0-100.0) | 50.0%  (-19.3-119.3) | 50.0%  (1.0-99.0) | 0.745 |
| **HD** (N=59) |  |  |  |  |  |  |  |
| CHE40 ^a^ | 32.2%  (20.3-44.1) | 62.5%  (38.8-86.2) | 25.0%  (0.5-49.5) | 27.3%  (1.0-53.6) | 9.1%  (-7.9-26.1) | 22.2%  (-4.9-49.4) | **0.046** |
| CHE10 ^b^ | 62.7%  (50.4-75.1) | 81.2%  (62.1-100.4) | 50.0%  (21.7-78.3) | 72.7%  (46.4-99.0) | 36.4%  (7.9-64.8) | 66.7%  (35.9-97.5) | 0.144 |

SSS, Social Security System

CKD15-60 chronic kidney disease with eGFR 15-60 ml/min/1.73m^2^, CKD<15 chronic kidney disease with eGFR<15 ml/min/1.73m^2^, PD peritoneal dialysis, HD hemodialysis

^a^:The percentage of households in which out-of-pocket payments for health care was at least 40% of household capacity to pay

^b^:The percentage of households in which out-of-pocket payments for health care was more than 10 % of households’ total consumption expenditure

**Table S5C** Socioeconomic status quintiles-specific proportion of CHE in CSMBS

| **Quintiles of socioeconomic**  **Status** | **Total**  (95% CI) | **1**  (95% CI) | **2**  (95% CI) | **3**  (95% CI) | **4**  (95% CI) | **5**  (95% CI) | **P-value** |
| --- | --- | --- | --- | --- | --- | --- | --- |
| **CKD15-60** (N=258) |  |  |  |  |  |  |  |
| CHE40 ^a^ | 10.9%  (7.1-14.6) | 17.6%  (7.2-28.1) | 20.3%  (10.1-30.6) | 5.1%  (-0.5-10.7) | 4.0%  (-1.4-9.4) | 5.1%  (-1.8-12.1) | **0.010** |
| CHE10 ^b^ | 19.8%  (14.9-24.6) | 29.4%  (16.9-41.9) | 30.5%  (18.8-42.3) | 16.9%  (7.4-26.5) | 12.0%  (3.0-21.0) | 5.1%  (-1.8-12.1) | **0.004** |
| **CKD<15** (N=90) |  |  |  |  |  |  |  |
| CHE40 ^a^ | 5.6%  (0.8-10.3) | 16.7%  (1.8-31.6) | 0.0%  (0.0-0.0) | 0.0%  (0.0-0.0) | 6.2%  (-5.6-18.1) | 0.0%  (0.0-0.0) | 0.079 |
| CHE10 ^b^ | 28.9%  (19.5-38.3) | 45.8%  (25.9-65.8) | 21.4%  (-0.1-42.9) | 23.5%  (3.4-43.7) | 18.8%  (-0.4-37.9) | 26.3%  (6.5-46.1) | 0.360 |
| **PD** (N=61) |  |  |  |  |  |  |  |
| CHE40 ^a^ | 24.6%  (13.8-35.4) | 70.0%  (41.6-98.4) | 36.4%  (7.9-64.8) | 23.1%  (0.2-46.0) | 0.0%  (0.0-0.0) | 5.6%  (-5.0-16.1) | **0.001** |
| CHE10 ^b^ | 42.6%  (30.2-55.0) | 90.0%  (71.4-108.6) | 45.5%  (16.0-74.9) | 38.5%  (12.0-64.9) | 0.0%  (0.0-0.0) | 38.9%  (16.4-61.4) | **0.002** |
| **HD** (N=166) |  |  |  |  |  |  |  |
| CHE40 ^a^ | 38.6%  (31.2-46.0) | 73.3%  (57.5-89.2) | 48.4%  (30.8-66.0) | 44.1%  (27.4-60.8) | 18.8%  (5.2-32.3) | 15.4%  (4.1-26.7) | **<0.001** |
| CHE10 ^b^ | 66.9%  (59.7-74.0) | 76.7%  (61.5-91.8) | 71.0%  (55.0-86.9) | 70.6%  (55.3-85.9) | 56.2%  (39.1-73.4) | 61.5%  (46.3-76.8) | 0.440 |

CSMBS, Civil Servant Monetary Benefit Scheme

CKD15-60 chronic kidney disease with eGFR 15-60 ml/min/1.73m^2^, CKD<15 chronic kidney disease with eGFR<15 ml/min/1.73m^2^, PD peritoneal dialysis, HD hemodialysis

^a^:The percentage of households in which out-of-pocket payments for health care was at least 40% of household capacity to pay

^b^:The percentage of households in which out-of-pocket payments for health care was more than 10 % of households’ total consumption expenditure

**Table S6** Proportion of Catastrophic Health Expenditure (CHE10) and impoverishment using poverty line by CKD groups

|  | **Total**  (95% CI) | **CKD15-60**  (95% CI) | **CKD<15**  (95% CI) | **PD**  (95% CI) | **HD**  (95% CI) | **P-value** |
| --- | --- | --- | --- | --- | --- | --- |
| **CHE10** ^a^ | | | | | |  |
| UCS | 33.9%  (29.9-37.9) | 15.7%  (9.9-21.4) | 19.4%  (12.0-26.9) | 40.5%*^$^  (33.5-47.6) | 67.0%*^$ #^  (57.5-76.5) | **< 0.001** |
| SSS | 46.8%  (37.4-56.2) | 8.3%  (-2.7-19.4) | 26.7%  (4.3-49.0) | 72.7%*^$^  (46.4-99.0) | 62.7% *^$^  (50.4-75.1) | **< 0.001** |
| CSMBS | 37.2%  (33.3-41.2) | 19.8%  (14.9-24.6) | 28.9%  (19.5-38.3) | 42.6%*^$^  (30.2-55.0) | 66.9%*^$ #^  (59.7-74.0) | **< 0.001** |
| **Medical impoverishment** | | | | | |  |
| **UCS** | | | | | |  |
| Pre-out-of-pocket impoverishment ^b^ | 1.9%  (0.7-3.0) | 1.3%  (-0.5-3.1) | 0.9%  (-0.9-2.7) | 2.2%  (0.1-4.3) | 3.2%  (-0.4-6.7) | 0.643 |
| Medical impoverishment ^c^ | 6.9%  (4.7-9.0) | 4.6%  (1.3-7.9) | 3.7%  (0.1-7.3) | 3.8%^$^  (1.0-6.5) | 20.2% *^$ #^  (12.1-28.3) | **< 0.001** |
| **SSS** | | | | | |  |
| Pre-out-of-pocket impoverishment ^b^ | 0.9%  (-0.9-2.7) | 0.0%  (0.0-0.0) | 0.0%  (0.0-0.0) | 0.0%  (0.0-0.0) | 1.7%  (-1.6-5.0) | 1.000 |
| Medical impoverishment ^c^ | 6.4%  (1.8-11.0) | 4.2%  (-3.8-12.2) | 0.0%  (0.0-0.0) | 0.0%  (0.0-0.0) | 10.2%  (2.5-17.9) | 0.591 |
| **CSMBS** | | | | | |  |
| Pre-out-of-pocket impoverishment ^b^ | 0.5%  (-0.1-1.1) | 0.8%  (-0.3-1.8) | 1.1%  (-1.1-3.3) | 0.0%  (0.0-0.0) | 0.0%  (0.0-0.0) | 0.591 |
| Medical impoverishment ^c^ | 3.1%  (1.7-4.6) | 2.3%  (0.5-4.2) | 0.0%  (0.0-0.0) | 3.3%  (-1.2-7.7) | 6.0%*^$ #^  (2.4-9.6) | 0.038 |

UCS, Universal Coverage Scheme. SSS, Social Security System. CSMBS, Civil Servant Monetary Benefit Scheme

CKD15-60 chronic kidney disease with eGFR 15-60 ml/min/1.73m^2^, CKD<15 chronic kidney disease with eGFR<15 ml/min/1.73m^2^, PD peritoneal dialysis, HD hemodialysis

^a^:The percentage of households in which out-of-pocket payments for health care was more than 10 % of households’ total consumption expenditure

^b^:The percentage of households in which total household expenditure was less than poverty line

^c^:The percentage of households in which total household expenditure after paying OOP for health, was less than poverty line

*P-value <0.05 vs CKD15-60, ^$^ P-value <0.05 vs CKD<15, ^#^ P-value <0.05 vs PD

**Table S7** Socioeconomic status quintiles-specific proportion of pre-out-of-pocket and impoverishment

**Table S7A** Socioeconomic status quintiles-specific proportion of pre-out-of-pocket and impoverishment in UCS

| **Quintiles of socioeconomic status** | **Total**  (95% CI) | **1**  (95% CI) | **2**  (95% CI) | **3**  (95% CI) | **4**  (95% CI) | **5**  (95% CI) | **P-value** |
| --- | --- | --- | --- | --- | --- | --- | --- |
| **CKD15-60** (N=153) |  |  |  |  |  |  |  |
| Pre-out-of-pocket impoverishment ^a^ | 18.3%  (12.2- 24.4) | 77.8%  (64.2-91.4) | 0.0%  (0.0-0.0) | 0.0%  (0.0-0.0) | 0.0%  (0.0-0.0) | 0.0%  (0.0-0.0) | **< 0.001** |
| Medical impoverishment ^b^ | 8.0%  (3.2-12.8) | 50.0%  (15.4-84.6) | 14.8%  (1.4-28.2) | 6.2%  (-2.1-14.6) | 0.0%  (0.0-0.0) | 0.0%  (0.0-0.0) | **< 0.001** |
| Pre-out-of-pocket impoverishment ^c^ | 1.3%  (-0.5-3.1) | 5.6%  (-1.9-13.0) | 0.0%  (0.0-0.0) | 0.0%  (0.0-0.0) | 0.0%  (0.0-0.0) | 0.0%  (0.0-0.0) | 0.198 |
| Medical impoverishment ^d^ | 4.6%  (1.3-7.9) | 11.1%  (0.8-21.4) | 3.7%  (-3.4-10.8) | 6.2%  (-2.1-14.6) | 0.0%  (0.0-0.0) | 0.0%  (0.0-0.0) | 0.161 |
| **CKD<15** (N=108) |  |  |  |  |  |  |  |
| Pre-out-of-pocket impoverishment ^a^ | 11.1%  (5.2-17.0) | 75.0%  (53.8-96.2) | 0.0%  (0.0-0.0) | 0.0%  (0.0-0.0) | 0.0%  (0.0-0.0) | 0.0%  (0.0-0.0) | **< 0.001** |
| Medical impoverishment ^b^ | 3.1%  (-0.4-6.6) | 50.0%  (1.0-99.0) | 0.0%  (0.0-0.0) | 3.8%  (-3.5-11.2) | 0.0%  (0.0-0.0) | 0.0%  (0.0-0.0) | **0.003** |
| Pre-out-of-pocket impoverishment ^c^ | 0.9%  (-0.9-2.7) | 6.2%  (-5.6-18.1) | 0.0%  (0.0-0.0) | 0.0%  (0.0-0.0) | 0.0%  (0.0-0.0) | 0.0%  (0.0-0.0) | 0.278 |
| Medical impoverishment ^d^ | 3.7%  (0.1-7.3) | 18.8%  (-0.4-37.9) | 0.0%  (0.0-0.0) | 3.8%  (-3.5-11.2) | 0.0%  (0.0-0.0) | 0.0%  (0.0-0.0) | **0.017** |
| **PD** (N=185) |  |  |  |  |  |  |  |
| Pre-out-of-pocket impoverishment ^a^ | 15.7%  (10.4-20.9) | 82.9%  (70.4-95.3) | 0.0%  (0.0-0.0) | 0.0%  (0.0-0.0) | 0.0%  (0.0-0.0) | 0.0%  (0.0-0.0) | **< 0.001** |
| Medical impoverishment ^b^ | 11.5%  (6.5-16.6) | 66.7%  (28.9-104.4) | 23.1%  (9.9-36.3) | 3.1%  (-2.9-9.2) | 4.8%  (-1.7-11.2) | 5.4%  (-1.9-12.7) | **< 0.001** |
| Pre-out-of-pocket impoverishment ^c^ | 2.2%  (0.1-4.3) | 11.4%  (0.9-22.0) | 0.0%  (0.0-0.0) | 0.0%  (0.0-0.0) | 0.0%  (0.0-0.0) | 0.0%  (0.0-0.0) | **0.002** |
| Medical impoverishment ^d^ | 3.8%  (1.0-6.5) | 14.3%  (2.7-25.9) | 0.0%  (0.0-0.0) | 0.0%  (0.0-0.0) | 2.4%  (-2.2-7.0) | 2.7%  (-2.5-7.9) | **0.011** |
| **HD** (N=94) |  |  |  |  |  |  |  |
| Pre-out-of-pocket impoverishment ^a^ | 19.1%  (11.2- 27.1) | 81.8%  (65.7-97.9) | 0.0%  (0.0-0.0) | 0.0%  (0.0-0.0) | 0.0%  (0.0-0.0) | 0.0%  (0.0-0.0) | **< 0.001** |
| Medical impoverishment ^b^ | 31.6%  (21.1- 42.0) | 100.0%  (100.0-100.0) | 61.1%  (38.6-83.6) | 27.8%  (7.1-48.5) | 15.0%  (-0.6-30.6) | 6.2%  (-5.6-18.1) | **< 0.001** |
| Pre-out-of-pocket impoverishment ^c^ | 3.2%  (-0.4-6.7) | 13.6%  (-0.7-28.0) | 0.0%  (0.0-0.0) | 0.0%  (0.0-0.0) | 0.0%  (0.0-0.0) | 0.0%  (0.0-0.0) | **0.036** |
| Medical impoverishment ^d^ | 20.2%  (12.1-28.3) | 31.8%  (12.4-51.3) | 33.3%  (11.6-55.1) | 16.7%  (-0.5-33.9) | 10.0%  (-3.1-23.1) | 6.2%  (-5.6-18.1) | 0.145 |

UCS, Universal Coverage Scheme

CKD15-60 chronic kidney disease with eGFR 15-60 ml/min/1.73m^2^, CKD<15 chronic kidney disease with eGFR<15 ml/min/1.73m^2^, PD peritoneal dialysis, HD hemodialysis

^a^:the percentage of households in which total household expenditure was less than computed subsistence expenditure

^b^:the percentage of households in which total household expenditure after paying OOP for health, was less than computed subsistence expenditure

^c^:the percentage of households in which total household expenditure was less than poverty line

^d^:the percentage of households in which total household expenditure after paying OOP for health, was less than poverty line

**Table S7B** Socioeconomic status quintiles-specific proportion of pre-out-of-pocket and impoverishment in SSS

| **Quintiles of socioeconomic status** | **Total**  (95% CI) | **1**  (95% CI) | **2**  (95% CI) | **3**  (95% CI) | **4**  (95% CI) | **5**  (95% CI) | **P-value** |
| --- | --- | --- | --- | --- | --- | --- | --- |
| **CKD15-60** (N=24) |  |  |  |  |  |  |  |
| Pre-out-of-pocket impoverishment ^a^ | 0.0%  (0.0-0.0) | 0.0%  (0.0-0.0) | 0.0%  (0.0-0.0) | 0.0%  (0.0-0.0) | 0.0%  (0.0-0.0) | 0.0%  (0.0-0.0) | - |
| Medical impoverishment ^b^ | 4.2%  (-3.8-12.2) | 0.0%  (0.0-0.0) | 0.0%  (0.0-0.0) | 16.7%  (-13.2-46.5) | 0.0%  (0.0-0.0) | 0.0%  (0.0-0.0) | 0.708 |
| Pre-out-of-pocket impoverishment ^c^ | 0.0%  (0.0-0.0) | 0.0%  (0.0-0.0) | 0.0%  (0.0-0.0) | 0.0%  (0.0-0.0) | 0.0%  (0.0-0.0) | 0.0%  (0.0-0.0) | - |
| Medical impoverishment ^d^ | 4.2%  (-3.8-12.2) | 0.0%  (0.0-0.0) | 0.0%  (0.0-0.0) | 16.7%  (-13.2-46.5) | 0.0%  (0.0-0.0) | 0.0%  (0.0-0.0) | 0.708 |
| **CKD<15**(N=15) |  |  |  |  |  |  |  |
| Pre-out-of-pocket impoverishment ^a^ | 0.0%  (0.0-0.0) | 0.0%  (0.0-0.0) | 0.0%  (0.0-0.0) | 0.0%  (0.0-0.0) | 0.0%  (0.0-0.0) | 0.0%  (0.0-0.0) | - |
| Medical impoverishment ^b^ | 0.0%  (0.0-0.0) | 0.0%  (0.0-0.0) | 0.0%  (0.0-0.0) | 0.0%  (0.0-0.0) | 0.0%  (0.0-0.0) | 0.0%  (0.0-0.0) | - |
| Pre-out-of-pocket impoverishment ^c^ | 0.0%  (0.0-0.0) | 0.0%  (0.0-0.0) | 0.0%  (0.0-0.0) | 0.0%  (0.0-0.0) | 0.0%  (0.0-0.0) | 0.0%  (0.0-0.0) | - |
| Medical impoverishment ^d^ | 0.0%  (0.0-0.0) | 0.0%  (0.0-0.0) | 0.0%  (0.0-0.0) | 0.0%  (0.0-0.0) | 0.0%  (0.0-0.0) | 0.0%  (0.0-0.0) | - |
| **PD** (N=11) |  |  |  |  |  |  |  |
| Pre-out-of-pocket impoverishment ^a^ | 0.09%^e^ | 0.0%  (0.0-0.0) | 0.0%  (0.0-0.0) | 0.0%  (0.0-0.0) | 0.0%  (0.0-0.0) | 0.0%  (0.0-0.0) | - |
| Medical impoverishment ^b^ | 0.0%  (0.0-0.0) | 0.0%  (0.0-0.0) | 0.0%  (0.0-0.0) | 0.0%  (0.0-0.0) | 0.0%  (0.0-0.0) | 0.0%  (0.0-0.0) | - |
| Pre-out-of-pocket impoverishment ^c^ | 0.0%  (0.0-0.0) | 0.0%  (0.0-0.0) | 0.0%  (0.0-0.0) | 0.0%  (0.0-0.0) | 0.0%  (0.0-0.0) | 0.0%  (0.0-0.0) | - |
| Medical impoverishment ^d^ | 0.0%  (0.0-0.0) | 0.0%  (0.0-0.0) | 0.0%  (0.0-0.0) | 0.0%  (0.0-0.0) | 0.0%  (0.0-0.0) | 0.0%  (0.0-0.0) | - |
| **HD** (N=59) |  |  |  |  |  |  |  |
| Pre-out-of-pocket impoverishment ^a^ | 8.5%  (1.4-15.6) | 31.2%  (8.5-54.0) | 0.0%  (0.0-0.0) | 0.0%  (0.0-0.0) | 0.0%  (0.0-0.0) | 0.0%  (0.0-0.0) | **0.007** |
| Medical impoverishment ^b^ | 24.1%  (12.7-35.5) | 54.5%  (25.1-84.0) | 16.7%  (-4.4-37.8) | 27.3%  (1.0-53.6) | 9.1%  (-7.9-26.1) | 11.1%  (-9.4-31.6) | 0.126 |
| Pre-out-of-pocket impoverishment ^c^ | 1.7%  (-1.6-5.0) | 6.2%  (-5.6-18.1) | 0.0%  (0.0-.0) | 0.0%  (0.0-0.0) | 0.0%  (0.0-0.0) | 0.0%  (0.0-0.0) | 1.000 |
| Medical impoverishment ^d^ | 10.2%  (2.5-17.9) | 25.0%  (3.8-46.2) | 0.0%  (0.0-0.0) | 9.1%  (-7.9-26.1) | 9.1%  (-7.9-26.1) | 0.0%  (0.0-0.0) | 0.217 |

SSS, Social Security System, CKD15-60 chronic kidney disease with eGFR 15-60 ml/min/1.73m^2^, CKD<15 chronic kidney disease with eGFR<15 ml/min/1.73m^2^,

PD peritoneal dialysis, HD hemodialysis

^a^:the percentage of households in which total household expenditure was less than computed subsistence expenditure

^b^:the percentage of households in which total household expenditure after paying OOP for health, was less than computed subsistence expenditure

^c^:the percentage of households in which total household expenditure was less than poverty line

^d^:the percentage of households in which total household expenditure after paying OOP for health, was less than poverty line, ^e^  1 patient with pre-OOP impoverishment

**Table S7C** Socioeconomic status quintiles-specific proportion of pre-out-of-pocket and impoverishment in CSMBS

| **Quintiles of socioeconomic status** | **Total**  (95% CI) | **1**  (95% CI) | **2**  (95% CI) | **3**  (95% CI) | **4**  (95% CI) | **5**  (95% CI) | **P-value** |
| --- | --- | --- | --- | --- | --- | --- | --- |
| **CKD15-60** (N=258) |  |  |  |  |  |  |  |
| Pre-out-of-pocket impoverishment ^a^ | 3.9%  (1.5-6.2) | 19.6%  (8.7-30.5) | 0.0%  (0.0-0.0) | 0.0%  (0.0-0.0) | 0.0%  (0.0-0.0) | 0.0%  (0.0-0.0) | **< 0.001** |
| Medical impoverishment ^b^ | 4.8%  (2.2-7.5) | 12.2%  (2.2-22.2) | 5.1%  (-0.5-10.7) | 3.4%  (-1.2-8.0) | 4.0%  (-1.4-9.4) | 0.0%  (0.0-0.0) | 0.167 |
| Pre-out-of-pocket impoverishment ^c^ | 0.8%  (-0.3-1.8) | 3.9%  (-1.4-9.2) | 0.0%  (0.0-0.0) | 0.0%  (0.0-0.0) | 0.0%  (0.0-0.0) | 0.0%  (0.0-0.0) | 0.098 |
| Medical impoverishment ^d^ | 2.3%  (0.5-4.2) | 2.0%  (-1.8-5.8) | 3.4%  (-1.2-8.0) | 3.4%  (-1.2-8.0) | 2.0%  (-1.9-5.9) | 0.0%  (0.0-0.0) | 0.904 |
| **CKD<15** (N=90) |  |  |  |  |  |  |  |
| Pre-out-of-pocket impoverishment ^a^ | 4.4%  (0.2-8.7) | 16.7%  (1.8-31.6) | 0.0%  (0.0-0.0) | 0.0%  (0.0-0.0) | 0.0%  (0.0-0.0) | 0.0%  (0.0-0.0) | **0.029** |
| Medical impoverishment ^b^ | 4.7%  (0.2-9.1) | 20.0%  (2.5-37.5) | 0.0%  (0.0-0.0) | 0.0%  (0.0-0.0) | 0.0%  (0.0-0.0) | 0.0%  (0.0-0.0) | **0.007** |
| Pre-out-of-pocket impoverishment ^c^ | 1.1%  (-1.1-3.3) | 4.2%  (-3.8-12.2) | 0.0%  (0.0-0.0) | 0.0 %  (0.0-0.0) | 0.0%  (0.0-0.0) | 0.0%  (0.0-0.0) | 1.000 |
| Medical impoverishment ^d^ | 0.0 %  (0.0-0.0) | 0.0%  (0.0-0.0) | 0.0%  (0.0-0.0) | 0.0%  (0.0-0.0) | 0.0%  (0.0-0.0) | 0.0%  (0.0-0.0) | - |
| **PD** (N=61) |  |  |  |  |  |  |  |
| Pre-out-of-pocket impoverishment ^a^ | 4.9%  (-0.5-10.3) | 30.0%  (1.6-58.4) | 0.0%  (0.0-0.0) | 0.0%  (0.0-0.0) | 0.0%  (0.0-0.0) | 0.0%  (0.0-0.0) | **0.006** |
| Medical impoverishment ^b^ | 6.9 %  (0.4-13.4) | 28.6%  (-4.9-62.0) | 9.1  (-7.9-26.1) | 7.7  (-6.8-22.2) | 0.0  (0.0-0.0) | 0.0  (0.0-0.0) | 0.090 |
| Pre-out-of-pocket impoverishment ^c^ | 0.0%  (0.0-0.0) | 0.0%  (0.0-0.0) | 0.0%  (0.0-0.0) | 0.0%  (0.0-0.0) | 0.0%  (0.0-0.0) | 0.0%  (0.0-0.0) | - |
| Medical impoverishment ^d^ | 3.3%  (-1.2-7.7) | 10.0%  (-8.6-28.6) | 9.1%  (-7.9-26.1) | 0.0%  (0.0-0.0) | 0.0%  (0.0-0.0) | 0.0%  (0.0-0.0) | 0.280 |
| **HD** (N=166) |  |  |  |  |  |  |  |
| Pre-out-of-pocket impoverishment ^a^ | 2.4%  (0.1-4.7) | 13.3%  (1.2-25.5) | 0.0%  (0.0-0.0) | 0.0%  (0.0; 0.0) | 0.0%  (0.0; 0.0) | 0.0%  (0.0-0.0) | **0.001** |
| Medical impoverishment ^b^ | 13.6%  (8.3-18.9) | 50.0%  (30.8-69.2) | 12.9%  (1.1-24.7) | 8.8%  (-0.7; 18.4) | 0.0%  (0.0; 0.0) | 5.1%  (-1.8-12.1) | **< 0.001** |
| Pre-out-of-pocket impoverishment ^c^ | 0.0%  (0.0-0.0) | 0.0%  (0.0-0.0) | 0.0%  (0.0-0.0) | 0.0%  (0.0; 0.0) | 0.0%  (0.0; 0.0) | 0.0%  (0.0-0.0) | - |
| Medical impoverishment ^d^ | 6.0%  (2.4-9.6) | 16.7%  (3.3-30.0) | 3.2%  (-3.0-9.4) | 5.9%  (-2.0; 13.8) | 0.0%  (0.0; 0.0) | 5.1%  (-1.8-12.1) | 0.095 |

CSMBS, Civil Servant Monetary Benefit Scheme

CKD15-60 chronic kidney disease with eGFR 15-60 ml/min/1.73m^2^, CKD<15 chronic kidney disease with eGFR<15 ml/min/1.73m^2^, PD peritoneal dialysis, HD hemodialysis

^a^:the percentage of households in which total household expenditure was less than computed subsistence expenditure

^b^:the percentage of households in which total household expenditure after paying OOP for health, was less than computed subsistence expenditure

^c^:the percentage of households in which total household expenditure was less than poverty line

^d^:the percentage of households in which total household expenditure after paying OOP for health, was less than poverty line

**Table S8** Multivariable adjusted probability of Catastrophic Health Expenditure (CHE)

| **Variables** | **CHE40** ^a,c^ | | **CHE10** ^b,c^ | |
| --- | --- | --- | --- | --- |
|  | **Average Probability** | **95% CI** | **Average Probability** | **95% CI** |
| CKD15-60#UCS | 0.076 | 0.042-0.109 | 0.149 | 0.116-0.183 |
| CKD15-60#SSS | 0.120 | -0.013-0.252 | 0.112 | -0.044-0.267 |
| CKD15-60#CSMBS | 0.096 | 0.059-0.134 | 0.183 | 0.130-0.236 |
| CKD<15#UCS | 0.088 | 0.052-0.125 | 0.187 | 0.126-0.248 |
| CKD<15#SSS | . | . | 0.306 | 0.159-0.453 |
| CKD<15#CSMBS | 0.049 | 0.003-0.094 | 0.266 | 0.142-0.390 |
| PD#UCS | 0.215 | 0.139-0.290 | 0.432 | 0.284-0.581 |
| PD#SSS | 0.582 | 0.319-0.845 | 0.753 | 0.547-0.959 |
| PD#CSMBS | 0.272 | 0.130-0.415 | 0.432 | 0.242-0.622 |
| HD#UCS | 0.527 | 0.366-0.687 | 0.694 | 0.588-0.800 |
| HD#SSS | 0.373 | 0.238-0.507 | 0.661 | 0.566-0.757 |
| HD#CSMBS | 0.403 | 0.284-0.522 | 0.668 | 0.588-0.748 |

UCS, Universal Coverage Scheme. SSS, Social Security System. CSMBS, Civil Servant Monetary Benefit Scheme

CKD15-60 chronic kidney disease with eGFR 15-60 ml/min/1.73m^2^, CKD<15 chronic kidney disease with eGFR<15 ml/min/1.73m^2^,

PD peritoneal dialysis, HD hemodialysis

^a^:The percentage of households in which out-of-pocket payments for health care was at least 40% of household capacity to pay.

^b^:The percentage of households in which out-of-pocket payments for health care was more than 10% of households’ total consumption expenditure

^c^ Adjusted with age, sex, diabetes, hypertension, cardiovascular disease, dyslipidemia, annual patient income, number of household members

95% CI, 95% Confidence Interval

**Table S9** Probability of incurring Catastrophic Health Expenditure (CHE) by regions from the modeling

| **Variables** | **CHE40** ^a,c^ | | **CHE10** ^b,c^ | |
| --- | --- | --- | --- | --- |
|  | **Average Probability** | **95% CI** | **Average Probability** | **95% CI** |
| **Regions** |  |  |  |  |
| Central | 0.297 | 0.231- 0.363 | 0.434 | 0.366 - 0.502 |
| North | 0.150 | 0.114 - 0.185 | 0.304 | 0.260 - 0.348 |
| Northeast | 0.228 | 0.164 - 0.291 | 0.363 | 0.307 - 0.420 |
| East | 0.122 | 0.103 - 0.141 | 0.313 | 0.283 - 0.343 |
| South | 0.178 | 0.127 - 0.230 | 0.377 | 0.356 - 0.397 |

^a^:The percentage of households in which out-of-pocket payments for health care was at least 40% of household capacity to pay.

^b^:The percentage of households in which out-of-pocket payments for health care was more than 10% of households’ total consumption expenditure

^c^ Adjusted with age, sex, CKD groups, health insurance scheme, diabetes, hypertension, cardiovascular disease, dyslipidemia, annual patient income, number of household members

95% CI, 95% Confidence Interval

**Figure S1** Flow of study

**
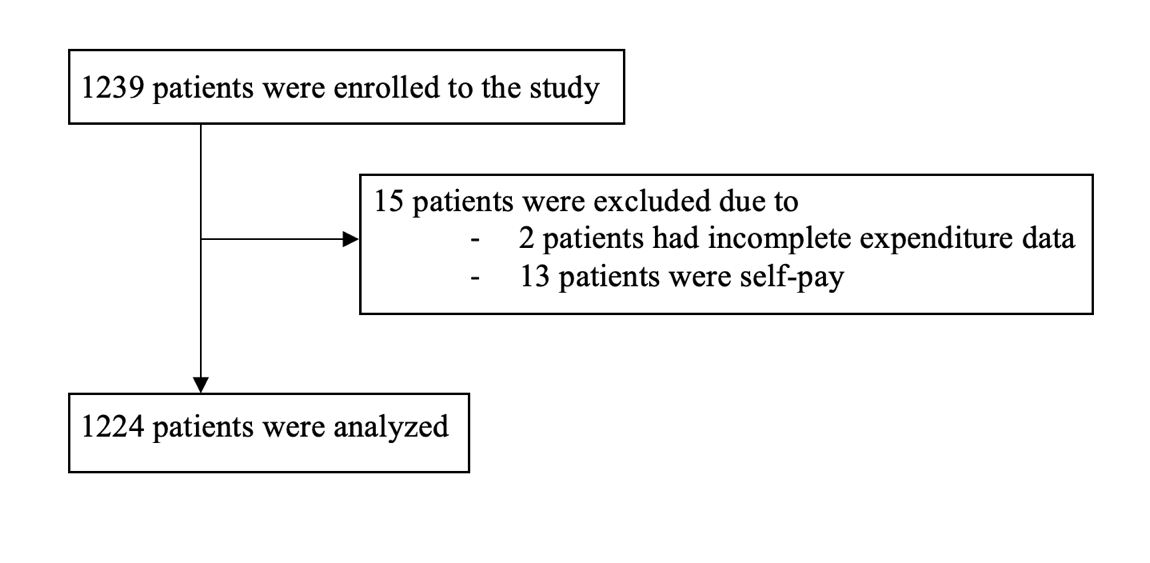
**

**Figure S2** Proportion of Catastrophic Health Expenditure (CHE40) ^a^ and impoverishment ^b,c^ according to CKD groups and health insurance schemes. (A) CHE40, (B) pre-OOP and medical impoverishment

**
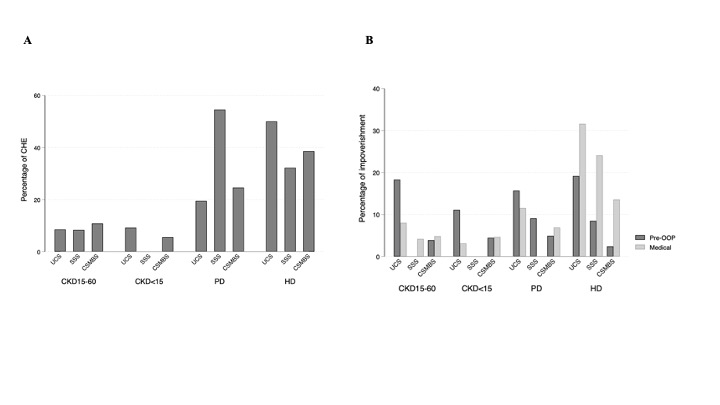
**

UCS, Universal Coverage Scheme. SSS, Social Security System. CSMBS, Civil Servant Monetary Benefit Scheme

CKD15-60 chronic kidney disease with eGFR 15-60 ml/min/1.73m^2^, CKD<15 chronic kidney disease with eGFR<15 ml/min/1.73m^2^, PD peritoneal dialysis, HD hemodialysis

^a^ The percentage of households in which out-of-pocket payments for health care was at least 40% of household capacity to pay

^b^ The percentage of households in which total household expenditure was less than computed subsistence expenditure

^c^ The percentage of households in which total household expenditure after paying OOP for health, was less than computed subsistence expenditure

**Figure S3** Socioeconomic status quintiles-specific proportion of pre-out-of-pocket (pre-OOP)^a^ and medical impoverishment^b^ .

(A) UCS, (B) SSS, (C) CSMBS.

**
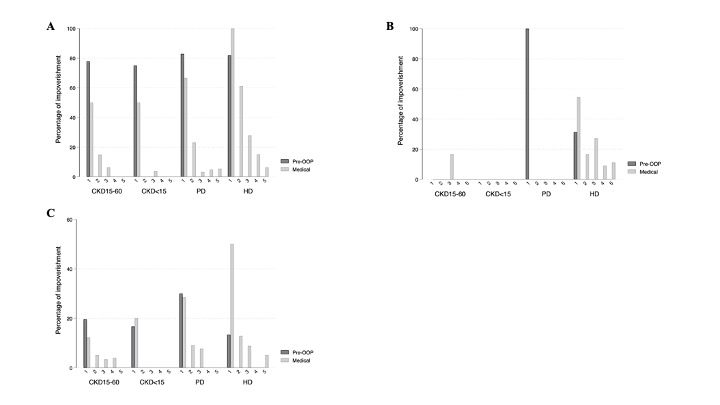
**

UCS, Universal Coverage Scheme. SSS, Social Security System. CSMBS, Civil Servant Monetary Benefit Scheme

CKD15-60 chronic kidney disease with eGFR 15-60 ml/min/1.73m^2^, CKD<15 chronic kidney disease with eGFR<15 ml/min/1.73m^2^, PD peritoneal dialysis, HD hemodialysis

^a^:the percentage of households in which total household expenditure was less than computed subsistence expenditure

^b^:the percentage of households in which total household expenditure after paying OOP for health, was less than computed subsistence expenditure

**Figure S4** Adjusted Probability of Catastrophic Health Expenditure (CHE40)^a^ by health insurance schemes and CKD groups

**
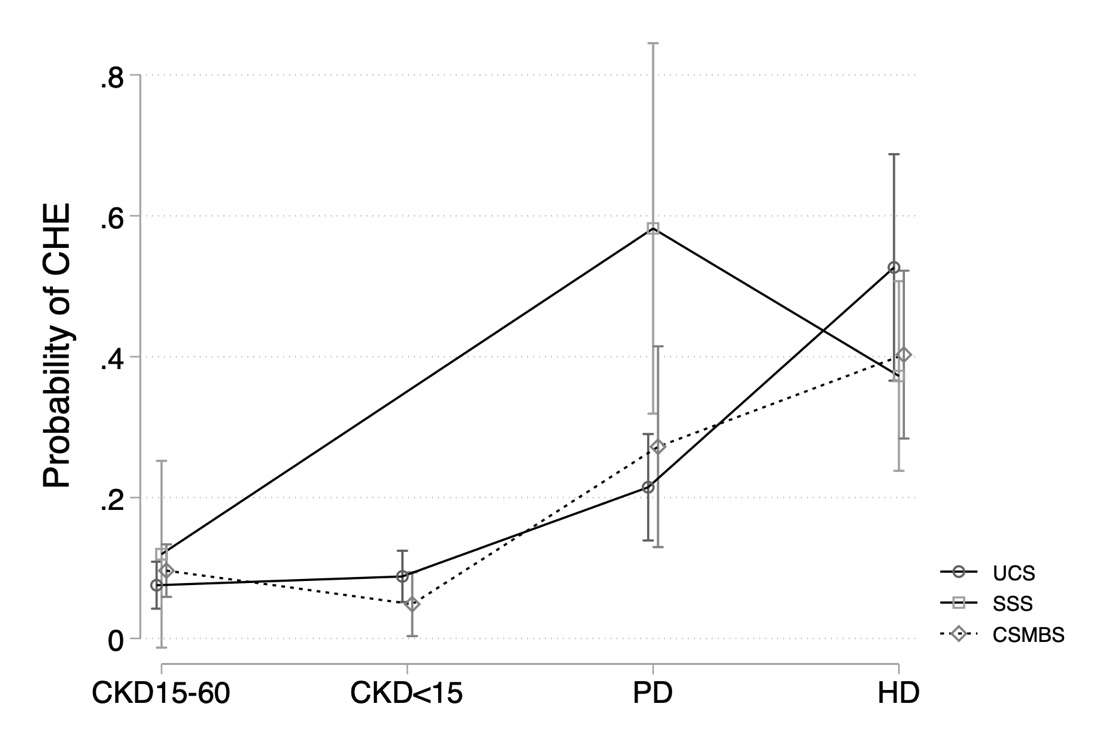
**

UCS, Universal Coverage Scheme. SSS, Social Security System. CSMBS, Civil Servant Monetary Benefit Scheme

CKD15-60 chronic kidney disease with eGFR 15-60 ml/min/1.73m^2^, CKD<15 chronic kidney disease with eGFR<15 ml/min/1.73m^2^,

PD peritoneal dialysis, HD hemodialysis

^a^ The percentage of households in which out-of-pocket payments for health care was at least 40% of household capacity to pay

Adjusted with age, sex, diabetes, hypertension, cardiovascular disease, dyslipidemia, annual patient income, number of household members
